# The impact of sleep disorders on glucose metabolism: endocrine and molecular mechanisms

**DOI:** 10.1186/s13098-015-0018-3

**Published:** 2015-03-24

**Authors:** Anne Briançon-Marjollet, Martin Weiszenstein, Marion Henri, Amandine Thomas, Diane Godin-Ribuot, Jan Polak

**Affiliations:** Université Grenoble Alpes, HP2, F-38041 Grenoble, Cedex France; INSERM U1042, F-38041 Grenoble, Cedex France; Centre for Research on Diabetes, Metabolism and Nutrition, Third Faculty of Medicine, Charles University, Prague, Czech Republic; 2nd Internal Medicine Department, University Hospital Kralovske Vinohrady, Prague, Czech Republic; Sports Medicine Department, Third Faculty of Medicine, Charles University in Prague, Ruska 87, Praha 10, 100 00 Czech Republic

**Keywords:** Sleep, Obstructive sleep apnea, Shift working, Intermittent hypoxia, Metabolic syndrome, Diabetes, Obesity, Insulin resistance

## Abstract

Modern lifestyle has profoundly modified human sleep habits. Sleep duration has shortened over recent decades from 8 to 6.5 hours resulting in chronic sleep deprivation. Additionally, irregular sleep, shift work and travelling across time zones lead to disruption of circadian rhythms and asynchrony between the master hypothalamic clock and pacemakers in peripheral tissues. Furthermore, obstructive sleep apnea syndrome (OSA), which affects 4 - 15% of the population, is not only characterized by impaired sleep architecture but also by repetitive hemoglobin desaturations during sleep. Epidemiological studies have identified impaired sleep as an independent risk factor for all cause of-, as well as for cardiovascular, mortality/morbidity. More recently, sleep abnormalities were causally linked to impairments in glucose homeostasis, metabolic syndrome and Type 2 Diabetes Mellitus (T2DM). This review summarized current knowledge on the metabolic alterations associated with the most prevalent sleep disturbances, i.e. short sleep duration, shift work and OSA. We have focused on various endocrine and molecular mechanisms underlying the associations between inadequate sleep quality, quantity and timing with impaired glucose tolerance, insulin resistance and pancreatic β-cell dysfunction. Of these mechanisms, the role of the hypothalamic-pituitary-adrenal axis, circadian pacemakers in peripheral tissues, adipose tissue metabolism, sympathetic nervous system activation, oxidative stress and whole-body inflammation are discussed. Additionally, the impact of intermittent hypoxia and sleep fragmentation (key components of OSA) on intracellular signaling and metabolism in muscle, liver, fat and pancreas are also examined. In summary, this review provides endocrine and molecular explanations for the associations between common sleep disturbances and the pathogenesis of T2DM.

## Introduction

Modern society, characterized by widespread use of electricity, demand for high performance at work, shift work, prolonged commute times and multiple leisure time activities, has significantly changed human sleep patterns. The average self-reported sleep duration has decreased from over 8 hours in the 1960’s to ≈ 6.5 hours in 2012, with 20–30% of middle aged Americans reporting sleep duration of less than 6 hours [1-7]. Similar patterns have been reported in other populations [8,9] and confirmed in studies employing actigraphy to objectively quantify sleep duration [10,11]. In addition to voluntary and work-related sleep restrictions, a variety of common sleep disorders such as insomnia and obstructive sleep apnea syndrome (OSA) contribute to impaired sleep in over 30% of adults [12].

Over the past decade, substantial evidence has accumulated showing that sleep disorders negatively impact not only cognitive functions and performance [13,14], but also cardiovascular morbidity and mortality [15-18]. More recently, it has been recognized that sleep is also causally related to the regulation of glucose homeostasis and appetite control and that impaired sleep contributes to the rising prevalence of obesity and Type 2 diabetes mellitus (T2DM) across the globe. In the following review our goal is to discuss possible mechanisms behind the epidemiological and experimental findings that document an independent role for sleep disturbances (including short sleep, shift work and OSA) in the development of glucose intolerance, insulin resistance and pancreatic endocrine dysfunction – impairments ultimately leading to T2DM. Although some epidemiological data suggest that excessively prolonged sleep might also be associated with metabolic impairments, we have chosen not to cover this topic here due to the lack of experimental tools, poor understanding of this association and the possible confounding effect of other chronic diseases.

## Review

### Physiological sleep

Sleep is an actively regulated, periodically occurring state of reduced consciousness, muscle relaxation and altered responsiveness to stimuli occurring in mammals, birds, reptiles, amphibians, fish and even in flies and worms. Based on patterns of brain electric activity, eye movements and skeletal muscle tone, human sleep is separated into distinct stages: non-rapid eye movement (NREM) sleep, which is further divided into stages according to sleep depth (stage 1, stage 2 and stage 3 also referred to as slow-wave sleep), and rapid eye movement (REM) sleep occurring every 60–90 minutes initially as short episodes, but which progressively increase in duration during the night.

Sleep onset and periodicity are precisely controlled processes determined by three major factors: a) circadian rhythms, b) homeostatic drive and c) emotional/cognitive inputs. Circadian rhythmicity of sleep onset is secured through the master pacemaker located in the hypothalamic suprachiasmatic nucleus (SCN) with projections into various regions in the brain participating in the regulation of sleep timing, behavioral and endocrine processes, food intake, physical activity and substrate metabolism. The intrinsic rhythm of these cells is independent of exogenous stimuli and is mediated by cell-autonomous periodic changes in gene expression and protein levels of transcriptional factors, such as Clock/Bmal (with a complex network of transcriptional-translational negative feedback loops). Adequate entrainment of SCN intrinsic oscillations with environmental light/dark cycles is mediated by direct projections from retina to the SCN. In contrast, homeostatic drive represents a sleep promoting mechanism with molecular actors which remain to be identified; its magnitude and thus the tendency to fall asleep increases with the duration of wakefulness and diminishes during sleep [19].

### Whole body metabolism during sleep

Contrasting physiological purposes of sleep and wakefulness are mirrored in substrate utilization and energy expenditure differences. During physiological sleep, which is harmonically entrained to environmental and behavioral zeitgebers (time cues), whole body energy expenditure drops by 15 - 35% with the lowest expenditure during slow-wave sleep and slightly higher during REM sleep [20]. Furthermore, glucose, lipid and protein turnover exhibits significant variability during the natural sleep/wake cycle that is independent of metabolic changes induced by food intake. For example, plasma glucose levels strongly follow the circadian pattern and progressively increase during sleep with the highest levels in the early morning [21-23]. Diurnal variations in glucose metabolism are mediated primarily through direct autonomic innervation of target organs from the SCN [21] and are independent of circulating insulin or glucagon levels [24,25]. In fact, SCN-driven autonomic output has been shown to regulate hepatic glucose output [21,26,27] and is most likely also involved in the reduction of skeletal muscle blood flow and decreased muscle glucose uptake during sleep [26-28]. In the anticipation of awakening, hepatic glucose output increases and contributes to the “dawn phenomenon” in healthy as well as diabetic subjects [28,29]. Additionally, decreased neuron activity during slow-wave sleep contributes to decreased glucose utilization by the brain during sleep [30]. Similar circadian sleep/wake oscillations have been described in lipid metabolism. Plasma triglycerides and fatty acids demonstrate strong circadian oscillations with progressively decreasing levels during sleep [31] when lipoprotein lipase (LPL) activity and fatty acid synthesis in adipose tissue are at their highest [28,32,33]. Some authors have reported a slight increase in plasma free fatty acids (FFA) and glycerol in the late stages of sleep, which has been attributed to central pacemaker activity as well as to the adipose tissue lipolysis promoting effects of growth hormone [28,34,35].

### Metabolic abnormalities in sleep disorders

**Figure 1:**
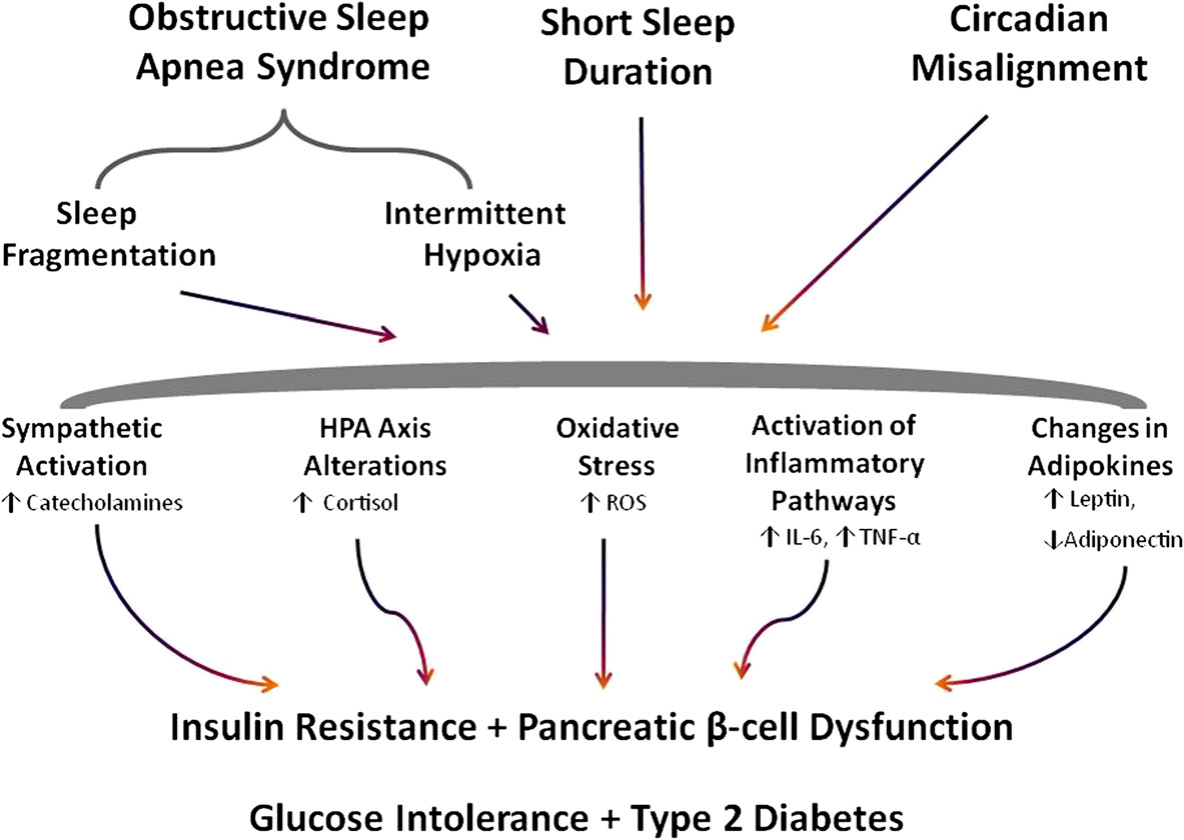
**Metabolic pathways linking sleep disorders with the development of Type 2 diabetes.** HPA (hypothalamic-pituitary-adrenal axis), ROS (reactive oxygen species), IL-6 (Interleukin-6), TNF-α (tumor necrosis factor-α).

Inadequate sleep duration together with misaligned or irregular sleep (e.g. during shift work) not only impairs cognitive performance [13,14,28] but are also associated with increased mortality and morbidity [15-18]. More recently, epidemiological and experimental studies have demonstrated that sleep quality and quantity are important determinants of whole-body metabolism. It has been suggested that impaired sleep might causally contribute to the T2DM and obesity epidemic via mechanisms depicted in Figure 1 and described further below.

### Short sleep duration

#### Models

The detrimental impact of short sleep duration on human health has been demonstrated in multiple studies including: a) cross-sectional studies, b) longitudinal epidemiological studies and c) experimental studies using variable severity and duration of sleep deprivation in human volunteers. Even though the physiological need for sleep significantly varies between individuals and is dependent on other factors such as age, epidemiological studies in adults typically consider 7–8 hours of sleep as “normal” sleep. In contrast, the definition of “short sleep” across studies is quite heterogeneous (from subjectively reported insufficient sleep to cut-off values for sleep duration set at < 5, < 6 or < 7 hours).

#### Evidence

Large-scale cross-sectional epidemiological studies conducted in various populations including adolescents, middle-aged and elderly subjects, hypertensive patients and pregnant women have convincingly and repeatedly demonstrated that self-reported sleep duration is associated with approximately doubled prevalence of T2DM or impaired glucose tolerance, particularly in women [36-47]. Importantly, cross-sectional associations between T2DM and short sleep are independent of other traditional risk factors for diabetes. Furthermore, subjectively perceived insufficient, poor or short sleep is associated with several pre-diabetic features such as fasting hyperglycemia, elevated postprandial glucose and insulin levels or indices of whole-body insulin resistance [39,46,48-56]. Finally, inadequate sleep has also been shown to be detrimental in patients who have already developed diabetes, since it negatively impacts glycemic control [45,47].

Interpretation of cross-sectional studies is inherently limited due to the uncertainty of causality and its eventual direction. In fact, several reports suggest that hyperglycemia, hyperinsulinemia and endocrine changes associated with T2DM can significantly influence sleep quality and quantity [57-59]. To better understand possible impact of sleep duration on metabolic homeostasis, observations based on prospective studies have proven to be very informative. In these studies, subjects with variable sleep habits and sleep duration, but free of diabetes, were followed for an extended period of time while newly diagnosed cases of T2DM were recorded. Results of these studies, which followed from 661 to 70,026 adults over 4 to 32 years [60-71] plus meta-analyses [2,72] fully support the relationship suggested in cross-sectional studies. After adjustments for known risk factors, subjects with short sleep duration have a higher relative risk (RR = 1.28 [1.03-1.60]) of developing T2DM compared to those with normal sleep times.

Additional support for the epidemiological evidence has been provided by experimental studies demonstrating that healthy human volunteers exposed to a rather severe paradigm of total sleep deprivation lasting from one to five days develop insulin resistance [73,74] and β-cell dysfunction [75]. As a consequence of insulin resistance combined with defects in insulin secretion, fasting and postprandial glucose levels were increased following sleep deprivation [75-79]. Although total sleep deprivation studies have provided important insights into the role of sleep in metabolic regulations, studies using milder paradigms, restricting sleep to 4–5 hours/night for several consecutive nights more closely mimic chronic sleep loss present in today’s lifestyles. Despite the heterogeneity of study designs, partially sleep-deprived subjects also exhibited impairments in numerous parameters of glucose tolerance and insulin sensitivity [80-87]. Interestingly, the metabolic profile observed after sleep restriction shared several similarities with T2DM, including decreased muscle glucose uptake, increased liver glucose output and pancreatic β-cell dysfunction [80,83,84,88].

#### Mechanisms

##### Endocrine

Despite the clear association between short sleep and metabolic impairments, the underlying endocrine and molecular mechanisms remain only partially elucidated. Among the suggested mechanisms, the causal role of the hypothalamo-pituitary-adrenal (HPA) axis and sympathetic activation are supported by the largest body of literature. Circulating cortisol, assessed either by 24 h profiles or by single measurements of evening cortisol levels, were elevated together with markers of sympathetic activation [81] and circulating catecholamines [86] after total or partial sleep deprivation [79-81,83,89,90] as well as in short sleepers [91]. In contrast, some studies reported impairments in glucose homeostasis in sleep restricted individuals along with unchanged cortisol and catecholamine levels [82-84,88]. The complexity of associated endocrine mechanisms can be further demonstrated by observations of elevated levels of pro-inflammatory cytokines, lower circulating testosterone, decreased thyroid stimulating hormone levels, impaired pulsatility of growth hormone secretion [81,92,93] and changes in adipokines secreted from adipose tissue [94-96] in short sleepers [97-103] as well as after sleep deprivation [104-107] (also reviewed in [108]).

##### Appetite regulation

Prospective and cross-sectional studies have also identified short sleep duration as an independent risk factor for weight gain and abdominal fat accumulation (as reviewed in [109,110]). Experimental evidence supports this association, since sleep-restricted subjects express a preference for fat and carbohydrate rich foods [111,112] and increase their daily caloric intake by ≈ 20% [112-116]. It is therefore reasonable to suggest that insufficient sleep stimulates food intake [117] and contributes to the development of obesity and metabolic syndrome. Furthermore, short sleep duration decreased the amount of fat overweight subjects lost during caloric restriction [118]. Within the complex network of factors regulating food intake [119], increased drive to eat in subjects exposed to sleep deprivation [81,111,118,120-122] or in patients with short sleep duration [39,123] has been linked to decreased leptin (limits food intake, secreted from adipose tissue) and elevated ghrelin (increases food intake, secreted mainly from the stomach) plasma levels. However, opposite or conflicting results have also been published [79,85,90,114,116,118,124,125] pointing to the role of other factors, e.g. decreased levels of anorexigenic peptide YY (PYY) [126]. In summary, it is safe to conclude that development of obesity, due to neuroendocrine changes, induced by inadequate sleep represents an additional independent risk factor for the development of metabolic abnormalities.

### Circadian misalignment and shift working

#### Peripheral tissue pacemakers

Non-traditional work schedules (including shift and night work) together with travel across time zones represent typical examples of circadian disruption. Under these circumstances, behavioral cues such as physical activity, food intake and sleep/wake cycling are misaligned with the autonomous timing of the central pacemaker located in the hypothalamic suprachiasmatic nucleus (SCN). Furthermore, cells of peripheral organs involved in metabolic control including the liver, adipose tissue and muscle express a functional network of pacemaker genes and exhibit circadian cycling in expression of these genes, similar to the autonomous circadian rhythmicity observed in the SCN. As a result, expression of hundreds of tissue-specific genes undergo circadian variation in peripheral tissues [127-130].

Because pacemakers in peripheral tissues do not get any direct information about the day/night cycle, other mechanisms have developed to secure harmonious synchronization of metabolic functions in peripheral tissues with the SCN (which receives information about light intensity through direct retinal projections). At the transcriptional level, entrainment of metabolic function in peripheral tissues could be mediated by glucocorticoids. Plasma levels of cortisol (or corticosterone in mice) exhibit a rigid circadian variability persisting even under conditions of experimental forced desynchronization [22]. Glucocorticoid synthesis and release is controlled by a peripheral clock-oscillator [131] entrained to the SCN via direct sympathetic innervation of the adrenals [132-134]. The resulting circadian oscillations in plasma glucocorticoid levels induce oscillations in gene expression in target tissues (e.g. the liver) by binding to the promoter region of the *Per* gene, which represents a key component of the peripheral pacemaker network in the liver, adipose tissue and skeletal muscle [135-140]. The unique feature of peripheral oscillators is that they can be entrained by external cues. For example, nutrition has been identified as a potent zeitgeber for peripheral pacemakers even when clock genes were deleted or the SCN damaged [141,142]. Similarly, physical activity and exercise have been shown to entrain peripheral oscillators especially in skeletal muscle [140].

Together with the transcriptional regulation of genes participating in peripheral pacemaker activity, metabolism in peripheral tissues can be synchronized with SCN via endocrine mechanisms. For example, metabolic responses to oscillations in plasma levels of melatonin (a hormone released from the pineal gland under direct SCN control) were documented in fat [143], muscle [144-146], the liver [147,148] and the pancreas [149]. Studies revealed that melatonin (or melatonin receptor agonist) administration improved glucose homeostasis through various mechanisms including enhanced glucose uptake, increased glucose-induced insulin secretion, improved insulin sensitivity or decreased liver gluconeogenesis in various animal models [144,145,147,148,150-159]. Melatonin or melatonin receptor agonists also increased glycogen synthesis in hepatocytes [147], limited fat accumulation in adipocytes [160] and even decreased adiposity in humans [161] and rats [162]. Additionally, growth hormone [163], thyroid stimulating hormone [164] and direct sympathetic innervation of peripheral tissues [165] also exhibits strong circadian rhythmicity and contributes to entrainment of peripheral pacemakers to the SCN and the metabolic needs of the whole organism.

#### Metabolic impact of shift working

The harmony between pacemakers located in the SCN and peripheral tissues and their synchronization with environmental and behavioral cycles such as light/dark cycle, sleep/wake cycle, food intake and physical activity is challenged by shift work and long-distance travel. Complete re-setting of the central biological pacemaker to a night shift work is extremely rare in humans, especially under rotating shift schedules [166-170]. Incomplete adaptation to irregular sleep pattern results in a significant misalignment between biological pacemakers and the living environment. Metabolically active tissues obtain environmental cues (e.g. nutrition and physical activity) at inappropriate “central” times or at an inappropriate times of their own pacemaker cycle. Alignment of central and peripheral pacemakers is important for survival and overall needs of the organism [171], while dyssynchrony results in serious consequences including increased cardiovascular mortality and morbidity [172-178] and higher risk of cancer (reviewed in [179,180]).

Shift work and circadian misalignment profoundly impair metabolic function and glucose homeostasis. Cross sectional and retrospective studies have found a higher prevalence of T2DM [181-183], glucose intolerance [183], insulin resistance [184,185] and metabolic syndrome in shift workers [186-188]. Furthermore, a meta-analysis of observational studies confirmed higher risk of T2DM in shift workers, particularly men, in all shift-working schedules except evening and mixed shifts [189]. Shift workers also gained more weight over time [190,191]. Causal effect of shift work in the development of metabolic abnormalities has been corroborated in several prospective studies conducted in men and women who engaged in shift work. The studies found a higher risk of developing metabolic syndrome [192-196] and T2DM [176,197-201], although some of these findings lost significance after being adjusted for changes in body weight. All of the above effects seem to resonate in patients who have already developed diabetes and seem to be especially affected by the negative consequences of shift work. Elevated HBA1C levels were reported in diabetics engaged in shift work and insufficient diabetes control was linked to the duration of shift work employment and the number of hours worked per shift [177,182,192,193,201].

#### Mechanisms

Studies using mice with whole-body or organ-selective mutations in pacemaker genes have demonstrated the crucial role of central and peripheral pacemakers in the regulation of glucose levels, glucose tolerance, insulin sensitivity, insulin secretion and food intake [202-208]. For example, liver-specific loss of the *Bmal* gene induces hypoglycemia and altered expression of genes involved in glucose metabolism [209], while the β-cell-specific *Bmal* gene deletion results in hyperglycemia and impaired glucose-induced insulin secretion [205] caused by excessive production of reactive oxygen species [203]. Similarly, mice fed under conditions of central and peripheral pacemaker misalignment gained more weight and developed insulin resistance [210]. Additionally, healthy human volunteers subjected to circadian misalignment exhibited decreased insulin sensitivity, impaired compensatory insulin secretion and increased CRP (C-reactive protein) despite preserved total sleep time [211]. These regulations were also observed in shift work subjects, where plasma glucose and insulin responses to a test meal were significantly higher when identical food was administered during the night as opposed to during the day [212]. In parallel, insulin resistance and hyperinsulinemia were observed in shift work individuals [212,213].

Prolonging the natural 24-hour day to 28 hours (or more) for several consecutive days provides an experimental tool to investigate the metabolic impact of circadian misalignment and shift work independently of the possible influence of circadian oscillations in metabolic and endocrine pathways. Using such an experimental paradigm of forced dyssynchrony in human volunteers resulted in elevated glucose and insulin levels along with impaired glucose tolerance and pancreatic β-cell dysfunction [22,84]. Additionally, circadian disruption accelerated diabetes development in diabetes-prone rats due to apoptosis of insulin secreting β-cells [214].

Recent studies have identified elevated FFA levels, decreased leptin levels and a disrupted cortisol rhythm as possible endocrine mechanisms contributing to the development of insulin resistance and β-cell dysfunction in shift workers and/or after circadian disruption. Furthermore, increased secretion of pro-inflammatory cytokines by macrophages has been reported after circadian disruption in mice [203,215], suggesting a putative mechanism for the overall pro-inflammatory activation typical of T2DM [216].

### Obstructive sleep apnea syndrome (OSA)

#### Metabolic impact of OSA

Obstructive Sleep Apnea Syndrome (OSA) is a common sleep disorder with a recognized prevalence of 3 - 7% in the general population. Its prevalence is actually increasing along with the prevalence of obesity, which represents the most important risk factor for OSA [217]. OSA is about twice as common in men than in women [218,219]. OSA is characterized by repeated obstructions of the upper airways during sleep, causing intermittent oxygen desaturations and arousals during sleep. OSA is widely recognized as an independent risk factor for cardiovascular diseases [220-222]. Moreover, a growing body of evidence suggests that OSA is also associated with a number of metabolic alterations such as dyslipidemia, insulin resistance, glucose intolerance and T2DM. This has been reviewed extensively in the last few years [223-226]. Several cross-sectional studies have shown that obstructive sleep apnea impaired glucose tolerance and/or insulin sensitivity, as measured by HOMA-IR, even after adjusting for BMI [227-230]. Overall, it has been reported that the prevalence of prediabetes, assessed by insulin resistance and glucose intolerance, was higher in OSA patients than in controls with estimates varying from 20 to 67% [231]. More importantly, the severity of nocturnal hypoxia in non-obese OSA patients was associated with insulin resistance [232], suggesting that the OSA-related hypoxia-reoxygenation sequences play a major role in this metabolic dysfunction.

In large longitudinal studies, such as the Wisconsin cohort or the Busselton Health Study, the authors were able to demonstrate that OSA was associated with a higher prevalence of T2DM over 2 to 11 year follow-up periods [233-236]. Meta-analysis of prospective studies confirmed that moderate to severe OSA increases the risk of development of T2DM by approx 60% [237]. Finally, based on the hypothesis that OSA can cause insulin resistance and diabetes, several studies have investigated whether OSA treatment by CPAP could reverse these deleterious effects. Uncontrolled studies examining the effect of CPAP on glucose tolerance and insulin sensitivity in OSA patients with or without diabetes have yielded mixed results, leading to no clear conclusion [231]. Of the nine randomized controlled trials that have examined the effect of CPAP (compared with sham- CPAP) on glucose metabolism, only four studies demonstrated beneficial effects for therapeutic CPAP [231]. However, it should be emphasized that overall compliance with CPAP therapy has been demonstrated to be rather limited [238] and could influence outcomes of published studies. Recent study showed that CPAP use limited to 4 hours/night (traditionally considered as the lower limit of “compliant” CPAP use) would not sufficiently treat REM-associated apneas and hypopneas, which are closely associated with HBA1c levels [239]. Only minor improvements in glucose control can thus be expected with 4 hours/night CPAP use and prolonged CPAP therapy (i.e. 7 hours/night) was calculated as necessary to achieve clinically significant improvements in HBA1c levels [239]. Similarly, changes in HBA1c levels were associated with CPAP use only in those T2DM patients, who used CPAP for more than 4 hours/night [240].

Non-Alcoholic Fatty Liver Disease (NAFLD), a prevalent liver disease in which fat excessively deposits in the liver, has been recently associated with OSA [241]. NAFLD is related to insulin resistance and is included among the clinical conditions associated with metabolic syndrome. Interestingly, it was suggested that OSA-induced intermittent hypoxia was associated with hepatic fibrosis and inflammation in both obese or non-obese patients [242-245]. Minville et al. further suggested that the severity of nocturnal hypoxia was independently associated with steatosis, and that pre-existing obesity exacerbated this effect [246]. These results were confirmed in pediatric OSA patients [247].

### Mechanisms

#### Intermittent hypoxia

##### Whole-body effects of intermittent hypoxia

Numerous studies have investigated the link between intermittent hypoxia, as a component of OSA, and insulin resistance. In several rodent models, chronic exposure to IH induced impaired glucose tolerance (GTT) [248-250], increased HOMA index [251-253] and impaired glucose clearance [254]. Moreover, acute IH exposure of healthy human volunteers resulted in a decrease in insulin sensitivity and glucose effectiveness (the ability of glucose itself to stimulate glucose uptake and suppress hepatic glucose production) [255].

**Figure 2:**
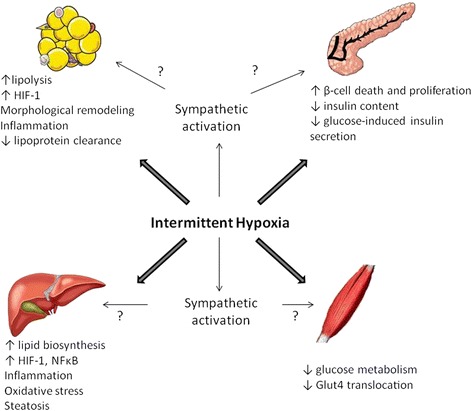
**Mechanisms linking intermittent hypoxia to impaired glucose metabolism.** Intermittent hypoxia acts on pancreatic insulin production and secretion as well as on insulin target organs such as adipose tissue, liver and skeletal muscle. These combined effects induce impaired glucose tolerance, insulin resistance and dyslipidemia. Intermittent hypoxia effects may be direct and/or mediated through the activation of the sympathetic nervous system. HIF-1α (hypoxia inducible factor 1-alpha), NF-κB (nuclear factor-κB), GLUT4 (glucose transporter type 4).

Overall, CIH appears to be responsible for carbohydrate dysregulation, nonetheless, the mechanisms involved remain unclear. In the following sections, we will review the consequences of IH on insulin target tissues, namely the liver, skeletal muscle, the pancreas and adipose tissue (summarized in Figure 2), with special emphasis on the molecular mechanisms involved, such as impaired lipid metabolism, inflammation, oxidative stress and sympathetic nervous system activation.

##### Impact of IH on the liver

*Structural damage.* Studies have demonstrated that CIH can induce liver damage and increase serum levels and activity of key liver enzymes such as serum aspartate aminotransferase, alanine aminotransferase and alkaline phosphatase in both mice and humans [251,256-258]. Several weeks of IH exposure resulted in liver steatosis, necrosis, inflammation with neutrophil accumulation and collagen deposits [256,257,259]. While triglyceride content is increased by CIH in lean and obese mice [251,257], hepatic cholesterol content appears to be depleted after 12 weeks of IH but was, by contrast, increased after 6 months in another study [257,258].

Exposure to IH led to increases in key lipid biosynthesis enzymes in the liver (SREBP-1, SCD-1 or HDL receptor) [260,261], at least partly mediated by HIF-1 transcription factor [262]. Glucose production is upregulated by IH, as supported by the observation of higher glycogen content [257,258], increased gene expression and protein levels of key gluconeogenic enzymes [250] and increased glucose output from isolated hepatocytes [250].

*Oxidative stress and inflammation.* Nitric oxide metabolites as well as liver iNOS levels have been shown to be increased by CIH along with reduced activity of liver antioxidant enzymes and DNA damage and apoptosis [256,263]. Moreover, IH resulted in increased lipid peroxidation and up-regulated p47phox expression and phosphorylation [264]. Pro-inflammatory cytokines, TNFα and Macrophage Inflammatory Protein 2 (MIP2) expression were unaffected by IH in lean mice but were increased in obese mice exposed to 4 weeks of IH [251]. However, longer periods of IH enhanced liver pro-inflammatory cytokines, such as IL-1β, IL-6 and MIP2 in lean mice, together with activation of the pro-inflammatory transcription factor NFkB [258]. Also, both HIF-1α and NFkB transcription factors have been shown to be up-regulated in the liver after 5 weeks of IH [263].

Overall, experimental evidence suggests that IH promotes liver injury and increases hepatocyte glucose output through several mechanisms. It should be noted that structural and functional lesions observed in IH-exposed livers resemble those of non-alcoholic fatty liver disease (NAFLD), a prevalent liver disease associated with OSA [246,265]. Therefore, apnea-related intermittent hypoxia appears to play a major role in OSA-related liver injury.

##### Impact of IH on skeletal muscle

As the principal insulin-sensitive organ, skeletal muscle is responsible for 80 to 90% of insulin-induced glucose uptake. However, probably because of the lack of consensus about the time-course of insulin resistance development, very few studies have focused on skeletal muscle metabolism in response to IH. It was suggested that IH-induced modifications of muscle metabolism were characterized by a decrease in creatine phosphate, citrate, alpha-ketoglutarate and glutamate content and by alterations in the anaerobic glycolytic pathway [266]*.* More recently, Liyori et al. observed a decrease in glucose metabolism in the soleus, a mostly oxidative muscle, using a mice model of CIH [252]. Finally, an alteration of the cytosolic-to-membrane translocation of GLUT4 that could provide an explanation for the development of IR in mice exposed to CIH [267]*.*

##### Impact of IH on the pancreas

Insight into the effects of IH on pancreatic function is of great importance for understanding mechanisms leading to impaired glucose homeostasis. Although many hypotheses have been proposed, only a few have been confirmed [268]. In severely obese adults, OSA was independently associated with an increase in basal pancreatic beta-cell function although glucose metabolism remained normal [269]. On the other hand, pancreatic insulin secretion was not affected in healthy volunteers exposed to IH, although insulin sensitivity and glucose effectiveness were diminished [255]. In mice exposed to CIH, beta-cell death as well as proliferation were observed [270,271]*.* Due to down-regulation of the enzyme prohormone convertase 1 (converting proinsulin to insulin), insulin content was decreased in islets from CIH-treated mice [272]. *In-vitro* cellular experiments have also suggested that IH exposure diminished glucose-induced insulin secretion due to down-regulation of CD38 gene transcription, which is involved in Ca^2+^ mobilization and thus insulin secretion [273]. Finally, studies in mice models using antioxidant strategies suggested that ROS are involved in IH-induced pancreatic damages [271,272].

##### Impact of IH on adipose tissue

Adipose tissue is generally recognized as a key player in insulin resistance. Free fatty acids (FFA) released by lipolysis of adipose tissue are able to induce insulin resistance through their effects on muscle, liver and adipose tissue itself (see [274] for review).

Results from several studies showed that IH can cause dyslipidemia through an increased FFA release [275-277] than can be normalized by oxygen supplementation in humans [276] and is accompanied with morphological and functional remodeling of the adipose tissue in mice [277]. Moreover, IH-induced dyslipidemia can also be related to decreased lipoprotein clearance due to the inhibition of lipoprotein lipase (LPL) mediated by HIF-1 and Angiopoietin-Like 4 (Angptl4) [249,277,278]. Finally, Iintermittent hypoxia down-regulates adiponectin in 3 T3-L1 adipocytes [279], which is a potent insulin-sensitizing hormone and increases adipose tissue production of resistin that can contribute to the development of insulin resistance through pro-inflammatory processes involving TNFα and IL-6 production [280].

#### Sympathetic nervous system activation

Healthy adults display lower sympathetic nervous system (SNS) activity during sleep than during wake-time. In contrast, OSA patients exhibit high level of sympathetic nervous system activity during both wake-time and sleep, accompanied by higher levels of circulating catecholamines [281,282]. Both human and animal models of IH reproduce this phenotype. Indeed, IH exposure has been shown to increase sympathetic nervous system activity in healthy humans [283] and in rodents [284,285]. Oxidative stress, increased HIF-1α signaling and decreased HIF-2 signaling as well as endothelin-1 have been proposed as key mechanisms in IH-induced SNS activation [286].

Increased sympathetic tone strongly impacts lipid and glucose metabolism, through circulating factors as well as neural innervation of the liver, pancreas, skeletal muscle and white adipose tissue [287-289], depicted in Figure 2. Adrenal epinephrine released during sympathetic activation triggers glucose production and impairs insulin secretion, thereby promoting insulin resistance [290]. Consistently, sympathetic nervous system inhibition by carotid body denervation abolished insulin resistance in a rat model of diet induced obesity [291] and abolished IH-induced fasting hyperglycemia and HOMA-IR elevation [292]. Moreover, epinephrine, and to a lesser degree norepinephrine, have been largely studied and acknowledged as crucial mediators of adipose tissue lipolysis [293,294] acting through several β-adrenoceptor subtypes [295,296]. It is therefore tempting to postulate that IH-induced lipolysis and insulin resistance might be mediated through sympathetic nervous system activation. Finally, sympathetic innervations could be involved in hepatic glucose release [297] and in muscle insulin resistance [298]. In human volunteers, a 5 hour IH exposure induces a decrease in insulin sensitivity along with an increase in sympathetic nervous system activity but to date no causal link has been demonstrated [255]. Even though using α-blockers or inhibiting epinephrine release by adrenal medullectomy improved glucose tolerance [299,300] and phentolamine treatment additionally prevented impairments in insulin secretion induced in mice by IH [299], the impact of IH on insulin sensitivity seems to be independent of autonomic activity as neither medullectomy, phentolamine treatment or administration of SNS blocking agent hexamethonium improved IH-induced insulin resistance in mice [252,299,300]. More studies are therefore needed to clarify the involvement of SNS activation in IH-induced metabolic dysregulation.

#### Sleep fragmentation

Apneic episodes, a cornerstone of OSA, are associated with bouts of increased brain activity (arousals) leading to repetitive partial or full awakenings and thus, sleep fragmentation [301]. Taking into consideration the multiple detrimental metabolic consequences of intermittent hypoxic exposure, the obvious question with important clinical and therapeutic implications has been asked: does sleep fragmentation per se, without concomitant hypoxemia contribute to the development of metabolic impairments observed in OSA?

Sleep fragmentation represents a situation where total sleep duration is preserved, but continuous sleep and its architecture is interrupted by internal (e.g. arousals in OSA) or external (e.g. auditory stimuli in experiments) factors. Experimental studies using sleep fragmentation paradigm showed, that disruption of sleep by auditory and mechanical stimuli for two to three nights decreased insulin sensitivity [302-304], which was not compensated by increased insulin secretion [303], suggesting that such exposures compromise fundaments of glucose homeostasis and induce impairments typical for pathogenesis of T2DM.

Epidemiological studies support the experimental evidence. Number of arousals was closely associated with fasting insulin levels and insulin resistance even after adjustments for age and severity of adiposity in young adults [301] and EEG cues of wake/sleep transitions were associated with decreased insulin sensitivity and impaired insulin secretion independently of age, sex, body mass index, sleep stages, the arousal index, and the apnea-hypopnea index [305]. Importantly, it was observed that sleep fragmentation exerts a negative impact in subjects with clinically manifested diabetes, as suggested by a community-based study investigating middle-age adults assessing sleep using wrist actigraphy which demonstrated that sleep fragmentation was associated with higher fasting glucose and insulin levels as well as with reduced insulin sensitivity in patients with T2DM, but not in non-diabetics [56]. Additionally, sleep of patients with T2DM is characterized by higher sleep fragmentation scores detected by wrist actigraphy [306].

Mechanisms linking sleep fragmentation to altered metabolic control probably include elevated night and morning cortisol levels [302,307] as well as sympathetic activation [302]. Additionally, sleep fragmentation is independently associated with increased adiposity [308] and less weight reduction during weight loss program [309]. Experiments performed in rodent models of acute and prolonged (2 weeks) sleep fragmentation confirmed increased adiposity, insulin resistance, hyperglycemia and impaired insulin secretion [310-312]. Additionally, animal demonstrated increased markers of inflammation and oxidative stress in adipose tissue, in parallel to elevated corticosteroid levels [310,313]. Sleep fragmentation in mice also induced changes in visceral adipose tissue transcriptome with modifications in signaling and metabolic pathways including glucose metabolism [314] and adipocyte differentiation [315], however it is not known, whether these changes happen also in humans. Besides endocrine effects, sleep fragmentation seems to also have epigenetic effects demonstrated by insulin resistance and increased body weight of offspring of pregnant dams exposed to sleep fragmentation [316].

Sleep fragmentation is typically accompanied by a reduction in slow-wave sleep duration, which represents another mechanism for impaired glucose metabolism. It has been proposed that slow-wave sleep is particularly important for metabolic homeostasis as selective suppression of slow-wave sleep (SWS), without perturbation of total sleep time, resulted in glucose intolerance, insulin resistance and impaired β-cell function [303]. Selective SWS suppression (but not REM sleep suppression) also elevated morning glucose and insulin levels and impaired post-prandial glucose homeostasis in healthy men [317]. Furthermore, sleep fragmentation impaired satiety perception, impaired insulin and glucagon-like peptide 1 response to meals [304] and reduced fat oxidation [318], making subjects prone to adipose tissue accumulation, especially under conditions of reduced satiety perception [304]. The importance of SWS in glucose homeostasis is further supported by cross-sectional studies documenting that SWS duration is strongly predicting glucose-induced insulin secretion in obese individuals [43] as well as by studies reporting shorter SWS in T2DM compared to nondiabetic subjects [58]. Importantly, duration of SWS was negatively associated with HbA1c levels also in T1DM patients [319], suggesting a global position of SWS in the regulation of glucose metabolism, independently of obesity or pathogenesis of T2DM. In contrast, REM sleep duration seems to be more related to energy homeostasis, as reduction in REM sleep is associated with obesity in children and adults [320-322], which could be at least partly explained by increased metabolic rate during REM sleep, which is lost with REM time reduction [323].

## Conclusions

In this review, we summarized the current knowledge of molecular and endocrine mechanisms underlying independent and possibly causal associations between short sleep, circadian rhythm disruption (as observed with shift working) and OSA with glucose intolerance, insulin resistance, impaired insulin secretion and ultimately T2DM. Based on the literature, it can be concluded that hypothalamic-pituitary-adrenal axis activation with increased circulating cortisol levels, misalignment between central and peripheral pacemakers, enhanced lipolysis and modified adipokine release in adipose tissue and intermittent hypoxia-induced sympathetic nervous system activation, generation of reactive oxygen species and the induction of a whole-body pro-inflammatory state are the most likely mediators. Several of these mechanisms represent potential drug targets, however future research is warranted to determine, whether targeting the above mentioned molecular regulations would provide metabolic benefit in patients with inappropriate sleep.

## References

[1] Kripke DF, Simons RN, Garfinkel L, Hammond EC (1979). Short and long sleep and sleeping pills. Is increased mortality associated?. Arch Gen Psychiatry.

[2] Cappuccio FP, D'Elia L, Strazzullo P, Miller MA (2010). Quantity and quality of sleep and incidence of type 2 diabetes: a systematic review and meta-analysis. Diabetes Care.

[3] Schoenborn CA, Adams PE (2010). Health behaviors of adults: United States, 2005–2007. Vital Health Stat 10.

[4] Krueger PM, Friedman EM (2009). Sleep duration in the United States: a cross-sectional population-based study. Am J Epidemiol.

[5] Centers for Disease Control and Prevention (2012). Short Sleep Duration Among Workers — United States, 2010. Morb Mortal Wkly Rep.

[6] National Sleep Foundation: 2012 Bedroom Poll. http://sleepfoundation.org/sleep-polls (2012). Accessed 1 Aug 2014

[7] Centers for Disease Control and Prevention (2011). Effect of Short Sleep Duration on Daily Activities - United States, 2005–2008. Morb Mortal Wkly Rep.

[8] Shankar A, Koh WP, Yuan JM, Lee HP, Yu MC (2008). Sleep duration and coronary heart disease mortality among Chinese adults in Singapore: a population-based cohort study. Am J Epidemiol.

[9] Tamakoshi A, Ohno Y (2004). Self-reported sleep duration as a predictor of all-cause mortality: results from the JACC study, Japan. Sleep.

[10] Lauderdale DS, Knutson KL, Yan LL, Rathouz PJ, Hulley SB, Sidney S (2006). Objectively measured sleep characteristics among early-middle-aged adults: the CARDIA study. Am J Epidemiol.

[11] Redline S, Kirchner HL, Quan SF, Gottlieb DJ, Kapur V, Newman A (2004). The effects of age, sex, ethnicity, and sleep-disordered breathing on sleep architecture. Arch Intern Med.

[12] National Sleep Foundation (2014). 2013 International Bedroom Poll.

[13] Stickgold R, Walker MP (2007). Sleep-dependent memory consolidation and reconsolidation 113. Sleep Med.

[14] Walker MP (2009). The role of sleep in cognition and emotion. Ann N Y Acad Sci.

[15] Punjabi NM, Caffo BS, Goodwin JL, Gottlieb DJ, Newman AB, O'Connor GT (2009). Sleep-disordered breathing and mortality: a prospective cohort study. PLoS Med.

[16] Chien KL, Chen PC, Hsu HC, Su TC, Sung FC, Chen MF (2010). Habitual sleep duration and insomnia and the risk of cardiovascular events and all-cause death: report from a community-based cohort. Sleep.

[17] Cappuccio FP, D'Elia L, Strazzullo P, Miller MA (2010). Sleep duration and all-cause mortality: a systematic review and meta-analysis of prospective studies. Sleep.

[18] Ikehara S, Iso H, Date C, Kikuchi S, Watanabe Y, Wada Y (2009). Association of sleep duration with mortality from cardiovascular disease and other causes for Japanese men and women: the JACC study. Sleep.

[19] Borbely AA, Tobler I (2011). Manifestations and functional implications of sleep homeostasis. Handb Clin Neurol.

[20] Katayose Y, Tasaki M, Ogata H, Nakata Y, Tokuyama K, Satoh M (2009). Metabolic rate and fuel utilization during sleep assessed by whole-body indirect calorimetry. Metabolism.

[21] Kalsbeek A, Perreau-Lenz S, Buijs RM (2006). A network of (autonomic) clock outputs. Chronobiol Int.

[22] Scheer FA, Hilton MF, Mantzoros CS, Shea SA (2009). Adverse metabolic and cardiovascular consequences of circadian misalignment. Proc Natl Acad Sci U S A.

[23] Bolli GB, De FP, De CS, Perriello G, Ventura MM, Calcinaro F (1984). Demonstration of a dawn phenomenon in normal human volunteers. Diabetes.

[24] La Fleur SE, Kalsbeek A, Wortel J, Buijs RM (1999). A suprachiasmatic nucleus generated rhythm in basal glucose concentrations. J Neuroendocrinol.

[25] Ruiter M, La Fleur SE, van HC, van d V, Kalsbeek A, Buijs RM (2003). The daily rhythm in plasma glucagon concentrations in the rat is modulated by the biological clock and by feeding behavior. Diabetes.

[26] Zoccoli G, Cianci T, Lenzi P, Franzini C (1992). Shivering during sleep: relationship between muscle blood flow and fiber type composition. Experientia.

[27] Morris CJ, Aeschbach D, Scheer FA (2012). Circadian system, sleep and endocrinology. Mol Cell Endocrinol.

[28] Clore JN, Nestler JE, Blackard WG (1989). Sleep-associated fall in glucose disposal and hepatic glucose output in normal humans. Putative signaling mechanism linking peripheral and hepatic events. Diabetes.

[29] Bolli GB, Gerich JE (1984). The "dawn phenomenon"–a common occurrence in both non-insulin-dependent and insulin-dependent diabetes mellitus. N Engl J Med.

[30] Van CE, Polonsky KS, Scheen AJ (1997). Roles of circadian rhythmicity and sleep in human glucose regulation. Endocr Rev.

[31] Pan X, Zhang Y, Wang L, Hussain MM (2010). Diurnal regulation of MTP and plasma triglyceride by CLOCK is mediated by SHP. Cell Metab.

[32] Bray MS, Young ME (2011). Regulation of fatty acid metabolism by cell autonomous circadian clocks: time to fatten up on information?. J Biol Chem.

[33] Gimble JM, Floyd ZE (2009). Fat circadian biology. J Appl Physiol (1985).

[34] Boyle PJ, Avogaro A, Smith L, Bier DM, Pappu AS, Illingworth DR (1992). Role of GH in regulating nocturnal rates of lipolysis and plasma mevalonate levels in normal and diabetic humans. Am J Physiol.

[35] Cooper BG, White JE, Ashworth LA, Alberti KG, Gibson GJ (1995). Hormonal and metabolic profiles in subjects with obstructive sleep apnea syndrome and the acute effects of nasal continuous positive airway pressure (CPAP) treatment. Sleep.

[36] Gottlieb DJ, Punjabi NM, Newman AB, Resnick HE, Redline S, Baldwin CM (2005). Association of sleep time with diabetes mellitus and impaired glucose tolerance. Arch Intern Med.

[37] Tuomilehto H, Peltonen M, Partinen M, Seppa J, Saaristo T, Korpi-Hyovalti E (2008). Sleep duration is associated with an increased risk for the prevalence of type 2 diabetes in middle-aged women - The FIN-D2D survey. Sleep Med.

[38] Najafian J, Mohamadifard N, Siadat ZD, Sadri G, Rahmati MR (2013). Association between sleep duration and diabetes mellitus: Isfahan Healthy Heart Program. Niger J Clin Pract.

[39] Chaput JP, Despres JP, Bouchard C, Tremblay A (2007). Association of sleep duration with type 2 diabetes and impaired glucose tolerance. Diabetologia.

[40] Fiorentini A, Valente R, Perciaccante A, Tubani L (2007). Sleep's quality disorders in patients with hypertension and type 2 diabetes mellitus. Int J Cardiol.

[41] Buxton OM, Marcelli E (2010). Short and long sleep are positively associated with obesity, diabetes, hypertension, and cardiovascular disease among adults in the United States. Soc Sci Med.

[42] Darukhanavala A, Booth JN, Bromley L, Whitmore H, Imperial J, Penev PD (2011). Changes in insulin secretion and action in adults with familial risk for type 2 diabetes who curtail their sleep. Diabetes Care.

[43] Koren D, Levitt Katz LE, Brar PC, Gallagher PR, Berkowitz RI, Brooks LJ (2011). Sleep architecture and glucose and insulin homeostasis in obese adolescents. Diabetes Care.

[44] Facco FL, Grobman WA, Kramer J, Ho KH, Zee PC (2010). Self-reported short sleep duration and frequent snoring in pregnancy: impact on glucose metabolism. Am J Obstet Gynecol.

[45] Knutson KL, Ryden AM, Mander BA, Van CE (2006). Role of sleep duration and quality in the risk and severity of type 2 diabetes mellitus. Arch Intern Med.

[46] Qiu C, Enquobahrie D, Frederick IO, Abetew D, Williams MA (2010). Glucose intolerance and gestational diabetes risk in relation to sleep duration and snoring during pregnancy: a pilot study. BMC Womens Health.

[47] Ohkuma T, Fujii H, Iwase M, Kikuchi Y, Ogata S, Idewaki Y (2013). Impact of sleep duration on obesity and the glycemic level in patients with type 2 diabetes: the Fukuoka Diabetes Registry. Diabetes Care.

[48] Jennings JR, Muldoon MF, Hall M, Buysse DJ, Manuck SB (2007). Self-reported sleep quality is associated with the metabolic syndrome. Sleep.

[49] Flint J, Kothare SV, Zihlif M, Suarez E, Adams R, Legido A (2007). Association between inadequate sleep and insulin resistance in obese children. J Pediatr.

[50] Matthews KA, Dahl RE, Owens JF, Lee L, Hall M (2012). Sleep duration and insulin resistance in healthy black and white adolescents. Sleep.

[51] Hung HC, Yang YC, Ou HY, Wu JS, Lu FH, Chang CJ (2013). The Association between Self-Reported Sleep Quality and Metabolic Syndrome. PLoS One.

[52] Hung HC, Yang YC, Ou HY, Wu JS, Lu FH, Chang CJ (2013). The relationship between impaired fasting glucose and self-reported sleep quality in a Chinese population. Clin Endocrinol (Oxf).

[53] Nakajima H, Kaneita Y, Yokoyama E, Harano S, Tamaki T, Ibuka E (2008). Association between sleep duration and hemoglobin A1c level. Sleep Med.

[54] Hall MH, Muldoon MF, Jennings JR, Buysse DJ, Flory JD, Manuck SB (2008). Self-reported sleep duration is associated with the metabolic syndrome in midlife adults. Sleep.

[55] Reutrakul S, Zaidi N, Wroblewski K, Kay HH, Ismail M, Ehrmann DA (2011). Sleep disturbances and their relationship to glucose tolerance in pregnancy. Diabetes Care.

[56] Knutson KL, Van CE, Zee P, Liu K, Lauderdale DS (2011). Cross-sectional associations between measures of sleep and markers of glucose metabolism among subjects with and without diabetes: the Coronary Artery Risk Development in Young Adults (CARDIA) Sleep Study. Diabetes Care.

[57] Song Y, Ye X, Ye L, Li B, Wang L, Hua Y (2013). Disturbed subjective sleep in chinese females with type 2 diabetes on insulin therapy. PLoS One.

[58] Pallayova M, Donic V, Gresova S, Peregrim I, Tomori Z (2010). Do differences in sleep architecture exist between persons with type 2 diabetes and nondiabetic controls?. J Diabetes Sci Technol.

[59] Nakanishi-Minami T, Kishida K, Funahashi T, Shimomura I (2012). Sleep-wake cycle irregularities in type 2 diabetics. Diabetol Metab Syndr.

[60] Ayas NT, White DP, Al-Delaimy WK, Manson JE, Stampfer MJ, Speizer FE (2003). A prospective study of self-reported sleep duration and incident diabetes in women. Diabetes Care.

[61] Nilsson PM, Roost M, Engstrom G, Hedblad B, Berglund G (2004). Incidence of diabetes in middle-aged men is related to sleep disturbances. Diabetes Care.

[62] Bjorkelund C, Bondyr-Carlsson D, Lapidus L, Lissner L, Mansson J, Skoog I (2005). Sleep disturbances in midlife unrelated to 32-year diabetes incidence: the prospective population study of women in Gothenburg. Diabetes Care.

[63] Mallon L, Broman JE, Hetta J (2005). High incidence of diabetes in men with sleep complaints or short sleep duration: a 12-year follow-up study of a middle-aged population. Diabetes Care.

[64] Yaggi HK, Araujo AB, McKinlay JB (2006). Sleep duration as a risk factor for the development of type 2 diabetes. Diabetes Care.

[65] Gangwisch JE, Heymsfield SB, Boden-Albala B, Buijs RM, Kreier F, Pickering TG (2007). Sleep duration as a risk factor for diabetes incidence in a large U.S. sample. Sleep.

[66] Beihl DA, Liese AD, Haffner SM (2009). Sleep duration as a risk factor for incident type 2 diabetes in a multiethnic cohort. Ann Epidemiol.

[67] Hayashino Y, Fukuhara S, Suzukamo Y, Okamura T, Tanaka T, Ueshima H (2007). Relation between sleep quality and quantity, quality of life, and risk of developing diabetes in healthy workers in Japan: the High-risk and Population Strategy for Occupational Health Promotion (HIPOP-OHP) Study. BMC Public Health.

[68] Kawakami N, Takatsuka N, Shimizu H (2004). Sleep disturbance and onset of type 2 diabetes. Diabetes Care.

[69] Meisinger C, Heier M, Loewel H (2005). Sleep disturbance as a predictor of type 2 diabetes mellitus in men and women from the general population. Diabetologia.

[70] Kita T, Yoshioka E, Satoh H, Saijo Y, Kawaharada M, Okada E (2012). Short sleep duration and poor sleep quality increase the risk of diabetes in Japanese workers with no family history of diabetes. Diabetes Care.

[71] von RA, Weikert C, Fietze I, Boeing H (2012). Association of sleep duration with chronic diseases in the European Prospective Investigation into Cancer and Nutrition (EPIC)-Potsdam study. PLoS One.

[72] Holliday EG, Magee CA, Kritharides L, Banks E, Attia J (2013). Short sleep duration is associated with risk of future diabetes but not cardiovascular disease: a prospective study and meta-analysis. PLoS One.

[73] Gonzalez-Ortiz M, Martinez-Abundis E, Balcazar-Munoz BR, Pascoe-Gonzalez S (2000). Effect of sleep deprivation on insulin sensitivity and cortisol concentration in healthy subjects. Diabetes Nutr Metab.

[74] VanHelder T, Symons JD, Radomski MW (1993). Effects of sleep deprivation and exercise on glucose tolerance. Aviat Space Environ Med.

[75] Benedict C, Hallschmid M, Lassen A, Mahnke C, Schultes B, Schioth HB (2011). Acute sleep deprivation reduces energy expenditure in healthy men. Am J Clin Nutr.

[76] Kuhn E, Brodan V, Brodanova M, Rysanek K (1969). Metabolic reflection of sleep deprivation. Act Nerv Super (Praha).

[77] Vondra K, Brodan V, Bass A, Kuhn E, Teisinger J, Andel M (1981). Effects of sleep deprivation on the activity of selected metabolic enzymes in skeletal muscle. Eur J Appl Physiol Occup Physiol.

[78] Wehrens SM, Hampton SM, Finn RE, Skene DJ (2010). Effect of total sleep deprivation on postprandial metabolic and insulin responses in shift workers and non-shift workers. J Endocrinol.

[79] Reynolds AC, Dorrian J, Liu PY, Van Dongen HP, Wittert GA, Harmer LJ (2012). Impact of five nights of sleep restriction on glucose metabolism, leptin and testosterone in young adult men. PLoS One.

[80] Spiegel K, Leproult R, Van CE (1999). Impact of sleep debt on metabolic and endocrine function. Lancet.

[81] Spiegel K, Leproult R, L'hermite-Baleriaux M, Copinschi G, Penev PD, Van CE (2004). Leptin levels are dependent on sleep duration: relationships with sympathovagal balance, carbohydrate regulation, cortisol, and thyrotropin. J Clin Endocrinol Metab.

[82] Schmid SM, Hallschmid M, Jauch-Chara K, Wilms B, Lehnert H, Born J (2011). Disturbed glucoregulatory response to food intake after moderate sleep restriction. Sleep.

[83] Buxton OM, Pavlova M, Reid EW, Wang W, Simonson DC, Adler GK (2010). Sleep restriction for 1 week reduces insulin sensitivity in healthy men. Diabetes.

[84] Buxton OM, Cain SW, O'Connor SP, Porter JH, Duffy JF, Wang W (2012). Adverse metabolic consequences in humans of prolonged sleep restriction combined with circadian disruption. Sci Transl Med.

[85] van Leeuwen WM, Hublin C, Sallinen M, Harma M, Hirvonen A, Porkka-Heiskanen T (2010). Prolonged sleep restriction affects glucose metabolism in healthy young men. Int J Endocrinol.

[86] Nedeltcheva AV, Kessler L, Imperial J, Penev PD (2009). Exposure to recurrent sleep restriction in the setting of high caloric intake and physical inactivity results in increased insulin resistance and reduced glucose tolerance. J Clin Endocrinol Metab.

[87] Robertson MD, Russell-Jones D, Umpleby AM, Dijk DJ (2013). Effects of three weeks of mild sleep restriction implemented in the home environment on multiple metabolic and endocrine markers in healthy young men. Metabolism.

[88] Donga E, van DM, van Dijk JG, Biermasz NR, Lammers GJ, van Kralingen KW (2010). A single night of partial sleep deprivation induces insulin resistance in multiple metabolic pathways in healthy subjects. J Clin Endocrinol Metab.

[89] Leproult R, Copinschi G, Buxton O, Van CE (1997). Sleep loss results in an elevation of cortisol levels the next evening. Sleep.

[90] Omisade A, Buxton OM, Rusak B (2010). Impact of acute sleep restriction on cortisol and leptin levels in young women. Physiol Behav.

[91] Kumari M, Badrick E, Ferrie J, Perski A, Marmot M, Chandola T (2009). Self-reported sleep duration and sleep disturbance are independently associated with cortisol secretion in the Whitehall II study. J Clin Endocrinol Metab.

[92] Leproult R, Van CE (2011). Effect of 1 week of sleep restriction on testosterone levels in young healthy men. JAMA.

[93] Spiegel K, Leproult R, Colecchia EF, L'hermite-Baleriaux M, Nie Z, Copinschi G (2000). Adaptation of the 24-h growth hormone profile to a state of sleep debt. Am J Physiol Regul Integr Comp Physiol.

[94] Hayes AL, Xu F, Babineau D, Patel SR (2011). Sleep duration and circulating adipokine levels. Sleep.

[95] Broussard JL, Ehrmann DA, Van CE, Tasali E, Brady MJ (2012). Impaired insulin signaling in human adipocytes after experimental sleep restriction: a randomized, crossover study. Ann Intern Med.

[96] Al-Disi D, Al-Daghri N, Khanam L, Al-Othman A, Al-Saif M, Sabico S (2010). Subjective sleep duration and quality influence diet composition and circulating adipocytokines and ghrelin levels in teen-age girls. Endocr J.

[97] Patel SR, Zhu X, Storfer-Isser A, Mehra R, Jenny NS, Tracy R (2009). Sleep duration and biomarkers of inflammation. Sleep.

[98] Ferrie JE, Kivimaki M, Akbaraly TN, Singh-Manoux A, Miller MA, Gimeno D (2013). Associations between change in sleep duration and inflammation: findings on C-reactive protein and interleukin 6 in the Whitehall II Study. Am J Epidemiol.

[99] Grandner MA, Buxton OM, Jackson N, Sands-Lincoln M, Pandey A, Jean-Louis G (2013). Extreme sleep durations and increased C-reactive protein: effects of sex and ethnoracial group. Sleep.

[100] Miller MA, Cappuccio FP (2013). Biomarkers of cardiovascular risk in sleep-deprived people. J Hum Hypertens.

[101] Martinez-Gomez D, Eisenmann JC, Gomez-Martinez S, Hill EE, Zapatera B, Veiga OL (2011). Sleep duration and emerging cardiometabolic risk markers in adolescents. The AFINOS study. Sleep Med.

[102] Miller MA, Kandala NB, Kivimaki M, Kumari M, Brunner EJ, Lowe GD (2009). Gender differences in the cross-sectional relationships between sleep duration and markers of inflammation: Whitehall II study. Sleep.

[103] Okun ML, Coussons-Read M, Hall M (2009). Disturbed sleep is associated with increased C-reactive protein in young women. Brain Behav Immun.

[104] Vgontzas AN, Zoumakis E, Bixler EO, Lin HM, Follett H, Kales A (2004). Adverse effects of modest sleep restriction on sleepiness, performance, and inflammatory cytokines. J Clin Endocrinol Metab.

[105] Shearer WT, Reuben JM, Mullington JM, Price NJ, Lee BN, Smith EO (2001). Soluble TNF-alpha receptor 1 and IL-6 plasma levels in humans subjected to the sleep deprivation model of spaceflight. J Allergy Clin Immunol.

[106] Haack M, Sanchez E, Mullington JM (2007). Elevated inflammatory markers in response to prolonged sleep restriction are associated with increased pain experience in healthy volunteers. Sleep.

[107] Meier-Ewert HK, Ridker PM, Rifai N, Regan MM, Price NJ, Dinges DF (2004). Effect of sleep loss on C-reactive protein, an inflammatory marker of cardiovascular risk. J Am Coll Cardiol.

[108] Grandner MA, Sands-Lincoln MR, Pak VM, Garland SN (2013). Sleep duration, cardiovascular disease, and proinflammatory biomarkers. Nat Sci Sleep.

[109] Morselli LL, Guyon A, Spiegel K (2012). Sleep and metabolic function. Pflugers Arch.

[110] Knutson KL (2010). Sleep duration and cardiometabolic risk: a review of the epidemiologic evidence. Best Pract Res Clin Endocrinol Metab.

[111] Spiegel K, Tasali E, Penev P, Van CE (2004). Brief communication: Sleep curtailment in healthy young men is associated with decreased leptin levels, elevated ghrelin levels, and increased hunger and appetite. Ann Intern Med.

[112] St-Onge MP, Roberts AL, Chen J, Kelleman M, O'Keeffe M, RoyChoudhury A (2011). Short sleep duration increases energy intakes but does not change energy expenditure in normal-weight individuals. Am J Clin Nutr.

[113] Brondel L, Romer MA, Nougues PM, Touyarou P, Davenne D (2010). Acute partial sleep deprivation increases food intake in healthy men. Am J Clin Nutr.

[114] Bosy-Westphal A, Hinrichs S, Jauch-Chara K, Hitze B, Later W, Wilms B (2008). Influence of partial sleep deprivation on energy balance and insulin sensitivity in healthy women. Obes Facts.

[115] Calvin AD, Carter RE, Adachi T, Macedo PG, Albuquerque FN, van der WC (2013). Effects of experimental sleep restriction on caloric intake and activity energy expenditure. Chest.

[116] Nedeltcheva AV, Kilkus JM, Imperial J, Kasza K, Schoeller DA, Penev PD (2009). Sleep curtailment is accompanied by increased intake of calories from snacks. Am J Clin Nutr.

[117] Chapman CD, Benedict C, Brooks SJ, Schioth HB (2012). Lifestyle determinants of the drive to eat: a meta-analysis. Am J Clin Nutr.

[118] Nedeltcheva AV, Kilkus JM, Imperial J, Schoeller DA, Penev PD (2010). Insufficient sleep undermines dietary efforts to reduce adiposity. Ann Intern Med.

[119] Suzuki K, Jayasena CN, Bloom SR (2012). Obesity and appetite control. Exp Diabetes Res.

[120] Guilleminault C, Powell NB, Martinez S, Kushida C, Raffray T, Palombini L (2003). Preliminary observations on the effects of sleep time in a sleep restriction paradigm. Sleep Med.

[121] St-Onge MP, O'Keeffe M, Roberts AL, RoyChoudhury A, Laferrere B (2012). Short sleep duration, glucose dysregulation and hormonal regulation of appetite in men and women. Sleep.

[122] Schmid SM, Hallschmid M, Jauch-Chara K, Born J, Schultes B (2008). A single night of sleep deprivation increases ghrelin levels and feelings of hunger in normal-weight healthy men. J Sleep Res.

[123] Taheri S, Lin L, Austin D, Young T, Mignot E (2004). Short sleep duration is associated with reduced leptin, elevated ghrelin, and increased body mass index. PLoS Med.

[124] Schmid SM, Hallschmid M, Jauch-Chara K, Wilms B, Benedict C, Lehnert H (2009). Short-term sleep loss decreases physical activity under free-living conditions but does not increase food intake under time-deprived laboratory conditions in healthy men. Am J Clin Nutr.

[125] Simpson NS, Banks S, Dinges DF (2010). Sleep restriction is associated with increased morning plasma leptin concentrations, especially in women. Biol Res Nurs.

[126] Magee CA, Huang X-F, Iverson DC, Caputi P (2009). Acute sleep restriction alters neuroendocrine hormones and appetite in healthy male adults. Sleep Biol Rhythm.

[127] Ptitsyn AA, Zvonic S, Conrad SA, Scott LK, Mynatt RL, Gimble JM (2006). Circadian clocks are resounding in peripheral tissues. PLoS Comput Biol.

[128] Zvonic S, Ptitsyn AA, Conrad SA, Scott LK, Floyd ZE, Kilroy G (2006). Characterization of peripheral circadian clocks in adipose tissues. Diabetes.

[129] Eckel-Mahan KL, Patel VR, Mohney RP, Vignola KS, Baldi P, Sassone-Corsi P (2012). Coordination of the transcriptome and metabolome by the circadian clock. Proc Natl Acad Sci U S A.

[130] Dallmann R, Viola AU, Tarokh L, Cajochen C, Brown SA (2012). The human circadian metabolome. Proc Natl Acad Sci U S A.

[131] Son GH, Chung S, Choe HK, Kim HD, Baik SM, Lee H (2008). Adrenal peripheral clock controls the autonomous circadian rhythm of glucocorticoid by causing rhythmic steroid production. Proc Natl Acad Sci U S A.

[132] Ishida A, Mutoh T, Ueyama T, Bando H, Masubuchi S, Nakahara D (2005). Light activates the adrenal gland: timing of gene expression and glucocorticoid release. Cell Metab.

[133] Otsuka T, Goto M, Kawai M, Togo Y, Sato K, Katoh K (2012). Photoperiod regulates corticosterone rhythms by altered adrenal sensitivity via melatonin-independent mechanisms in Fischer 344 rats and C57BL/6 J mice. PLoS One.

[134] Wotus C, Lilley TR, Neal AS, Suleiman NL, Schmuck SC, Smarr BL (2013). Forced desynchrony reveals independent contributions of suprachiasmatic oscillators to the daily plasma corticosterone rhythm in male rats. PLoS One.

[135] Gomez-Abellan P, ez-Noguera A, Madrid JA, Lujan JA, Ordovas JM, Garaulet M (2012). Glucocorticoids affect 24 h clock genes expression in human adipose tissue explant cultures. PLoS One.

[136] Pezuk P, Mohawk JA, Wang LA, Menaker M (2012). Glucocorticoids as entraining signals for peripheral circadian oscillators. Endocrinology.

[137] So AY, Bernal TU, Pillsbury ML, Yamamoto KR, Feldman BJ (2009). Glucocorticoid regulation of the circadian clock modulates glucose homeostasis. Proc Natl Acad Sci U S A.

[138] Almon RR, Yang E, Lai W, Androulakis IP, Ghimbovschi S, Hoffman EP (2008). Relationships between circadian rhythms and modulation of gene expression by glucocorticoids in skeletal muscle. Am J Physiol Regul Integr Comp Physiol.

[139] Oishi K, Amagai N, Shirai H, Kadota K, Ohkura N, Ishida N (2005). Genome-wide expression analysis reveals 100 adrenal gland-dependent circadian genes in the mouse liver. DNA Res.

[140] Zambon AC, McDearmon EL, Salomonis N, Vranizan KM, Johansen KL, Adey D (2003). Time- and exercise-dependent gene regulation in human skeletal muscle. Genome Biol.

[141] Storch KF, Weitz CJ (2009). Daily rhythms of food-anticipatory behavioral activity do not require the known circadian clock. Proc Natl Acad Sci U S A.

[142] Sheward WJ, Maywood ES, French KL, Horn JM, Hastings MH, Seckl JR (2007). Entrainment to feeding but not to light: circadian phenotype of VPAC2 receptor-null mice. J Neurosci.

[143] onso-Vale MI, Andreotti S, Mukai PY, Borges-Silva C, Peres SB, Cipolla-Neto J (2008). Melatonin and the circadian entrainment of metabolic and hormonal activities in primary isolated adipocytes. J Pineal Res.

[144] Contreras-Alcantara S, Baba K, Tosini G (2010). Removal of melatonin receptor type 1 induces insulin resistance in the mouse. Obesity (Silver Spring).

[145] Sartori C, Dessen P, Mathieu C, Monney A, Bloch J, Nicod P (2009). Melatonin improves glucose homeostasis and endothelial vascular function in high-fat diet-fed insulin-resistant mice. Endocrinology.

[146] Ha E, Yim SV, Chung JH, Yoon KS, Kang I, Cho YH (2006). Melatonin stimulates glucose transport via insulin receptor substrate-1/phosphatidylinositol 3-kinase pathway in C2C12 murine skeletal muscle cells. J Pineal Res.

[147] Shieh JM, Wu HT, Cheng KC, Cheng JT (2009). Melatonin ameliorates high fat diet-induced diabetes and stimulates glycogen synthesis via a PKCzeta-Akt-GSK3beta pathway in hepatic cells. J Pineal Res.

[148] Faria JA, Kinote A, Ignacio-Souza LM, de Araujo TM, Razolli DS, Doneda DL (2013). Melatonin acts through MT1/MT2 receptors to activate hypothalamic Akt and suppress hepatic gluconeogenesis in rats. Am J Physiol Endocrinol Metab.

[149] Bahr I, Muhlbauer E, Albrecht E, Peschke E (2012). Evidence of the receptor-mediated influence of melatonin on pancreatic glucagon secretion via the Galphaq protein-coupled and PI3K signaling pathways. J Pineal Res.

[150] Park JH, Shim HM, Na AY, Bae KC, Bae JH, Im SS (2014). Melatonin prevents pancreatic beta-cell loss due to glucotoxicity: the relationship between oxidative stress and endoplasmic reticulum stress. J Pineal Res.

[151] Zanuto R, Siqueira-Filho MA, Caperuto LC, Bacurau RF, Hirata E, Peliciari-Garcia RA (2013). Melatonin improves insulin sensitivity independently of weight loss in old obese rats. J Pineal Res.

[152] Korkmaz GG, Uzun H, Cakatay U, Aydin S (2012). Melatonin ameliorates oxidative damage in hyperglycemia-induced liver injury. Clin Invest Med.

[153] Cuesta S, Kireev R, Garcia C, Rancan L, Vara E, Tresguerres JA (2013). Melatonin can improve insulin resistance and aging-induced pancreas alterations in senescence-accelerated prone male mice (SAMP8). Age (Dordr).

[154] de Oliveira AC, Andreotti S, Farias TS, Torres-Leal FL, de Proenca AR, Campana AB (2012). Metabolic disorders and adipose tissue insulin responsiveness in neonatally STZ-induced diabetic rats are improved by long-term melatonin treatment. Endocrinology.

[155] Kitagawa A, Ohta Y, Ohashi K (2012). Melatonin improves metabolic syndrome induced by high fructose intake in rats. J Pineal Res.

[156] Agil A, Rosado I, Ruiz R, Figueroa A, Zen N, Fernandez-Vazquez G (2012). Melatonin improves glucose homeostasis in young Zucker diabetic fatty rats. J Pineal Res.

[157] Nogueira TC, Lellis-Santos C, Jesus DS, Taneda M, Rodrigues SC, Amaral FG (2011). Absence of melatonin induces night-time hepatic insulin resistance and increased gluconeogenesis due to stimulation of nocturnal unfolded protein response. Endocrinology.

[158] Bertuglia S, Reiter RJ (2009). Melatonin reduces microvascular damage and insulin resistance in hamsters due to chronic intermittent hypoxia. J Pineal Res.

[159] Nishida S, Segawa T, Murai I, Nakagawa S (2002). Long-term melatonin administration reduces hyperinsulinemia and improves the altered fatty-acid compositions in type 2 diabetic rats via the restoration of Delta-5 desaturase activity. J Pineal Res.

[160] Wang PP, She MH, He PP, Chen WJ, Laudon M, Xu XX (2013). Piromelatine decreases triglyceride accumulation in insulin resistant 3 T3-L1 adipocytes: role of ATGL and HSL. Biochimie.

[161] Borba CP, Fan X, Copeland PM, Paiva A, Freudenreich O, Henderson DC (2011). Placebo-controlled pilot study of ramelteon for adiposity and lipids in patients with schizophrenia. J Clin Psychopharmacol.

[162] She M, Deng X, Guo Z, Laudon M, Hu Z, Liao D (2009). NEU-P11, a novel melatonin agonist, inhibits weight gain and improves insulin sensitivity in high-fat/high-sucrose-fed rats. Pharmacol Res.

[163] Surya S, Symons K, Rothman E, Barkan AL (2006). Complex rhythmicity of growth hormone secretion in humans. Pituitary.

[164] Patel YC, Alford FP, Burger HG (1972). The 24-hour plasma thyrotrophin profile. Clin Sci.

[165] Cailotto C, Lei J, van der V, van HC, van Eden CG, Kalsbeek A (2009). Effects of nocturnal light on (clock) gene expression in peripheral organs: a role for the autonomic innervation of the liver. PLoS One.

[166] Dumont M, Lanctot V, Cadieux-Viau R, Paquet J (2012). Melatonin production and light exposure of rotating night workers. Chronobiol Int.

[167] Smith MR, Eastman CI (2012). Shift work: health, performance and safety problems, traditional countermeasures, and innovative management strategies to reduce circadian misalignment. Nat Sci Sleep.

[168] Grundy A, Sanchez M, Richardson H, Tranmer J, Borugian M, Graham CH (2009). Light intensity exposure, sleep duration, physical activity, and biomarkers of melatonin among rotating shift nurses. Chronobiol Int.

[169] Folkard S (2008). Do permanent night workers show circadian adjustment? A review based on the endogenous melatonin rhythm. Chronobiol Int.

[170] Roden M, Koller M, Pirich K, Vierhapper H, Waldhauser F (1993). The circadian melatonin and cortisol secretion pattern in permanent night shift workers. Am J Physiol.

[171] Buijs RM, Escobar C, Swaab DF (2013). The circadian system and the balance of the autonomic nervous system. Handb Clin Neurol.

[172] Vyas MV, Garg AX, Iansavichus AV, Costella J, Donner A, Laugsand LE (2012). Shift work and vascular events: systematic review and meta-analysis. BMJ.

[173] Wang XS, Armstrong ME, Cairns BJ, Key TJ, Travis RC (2011). Shift work and chronic disease: the epidemiological evidence. Occup Med (Lond).

[174] Ha J, Kim SG, Paek D, Park J (2011). The Magnitude of Mortality from Ischemic Heart Disease Attributed to Occupational Factors in Korea - Attributable Fraction Estimation Using Meta-analysis. Saf Health Work.

[175] Tuchsen F, Hannerz H, Burr H (2006). A 12 year prospective study of circulatory disease among Danish shift workers. Occup Environ Med.

[176] Karlsson B, Alfredsson L, Knutsson A, Andersson E, Toren K (2005). Total mortality and cause-specific mortality of Swedish shift- and dayworkers in the pulp and paper industry in 1952–2001. Scand J Work Environ Health.

[177] Kawachi I, Colditz GA, Stampfer MJ, Willett WC, Manson JE, Speizer FE (1995). Prospective study of shift work and risk of coronary heart disease in women. Circulation.

[178] Knutsson A, Akerstedt T, Jonsson BG, Orth-Gomer K (1986). Increased risk of ischaemic heart disease in shift workers. Lancet.

[179] Evans JA, Davidson AJ (2013). Health consequences of circadian disruption in humans and animal models. Prog Mol Biol Transl Sci.

[180] Haus EL, Smolensky MH (2013). Shift work and cancer risk: potential mechanistic roles of circadian disruption, light at night, and sleep deprivation. Sleep Med Rev.

[181] Guo Y, Liu Y, Huang X, Rong Y, He M, Wang Y (2013). The effects of shift work on sleeping quality, hypertension and diabetes in retired workers. PLoS One.

[182] Monk TH, Buysse DJ (2013). Exposure to shift work as a risk factor for diabetes. J Biol Rhythms.

[183] Mikuni E, Ohoshi T, Hayashi K, Miyamura K (1983). Glucose intolerance in an employed population. Tohoku J Exp Med.

[184] Nagaya T, Yoshida H, Takahashi H, Kawai M (2002). Markers of insulin resistance in day and shift workers aged 30–59 years. Int Arch Occup Environ Health.

[185] Karlsson B, Knutsson A, Lindahl B (2001). Is there an association between shift work and having a metabolic syndrome? Results from a population based study of 27,485 people. Occup Environ Med.

[186] Puttonen S, Viitasalo K, Harma M (2012). The relationship between current and former shift work and the metabolic syndrome. Scand J Work Environ Health.

[187] Esquirol Y, Bongard V, Mabile L, Jonnier B, Soulat JM, Perret B (2009). Shift work and metabolic syndrome: respective impacts of job strain, physical activity, and dietary rhythms. Chronobiol Int.

[188] Karlsson BH, Knutsson AK, Lindahl BO, Alfredsson LS (2003). Metabolic disturbances in male workers with rotating three-shift work. Results of the WOLF study. Int Arch Occup Environ Health.

[189] Gan Y, Yang C, Tong X, Sun H, Cong Y, Yin X (2015). Shift work and diabetes mellitus: a meta-analysis of observational studies. Occup Environ Med.

[190] Suwazono Y, Dochi M, Sakata K, Okubo Y, Oishi M, Tanaka K (2008). A longitudinal study on the effect of shift work on weight gain in male Japanese workers. Obesity (Silver Spring).

[191] Niedhammer I, Lert F, Marne MJ (1996). Prevalence of overweight and weight gain in relation to night work in a nurses' cohort. Int J Obes Relat Metab Disord.

[192] Kawada T, Otsuka T (2014). Effect of shift work on the development of metabolic syndrome after 3 years in Japanese male workers. Arch Environ Occup Health.

[193] Suwazono Y, Uetani M, Oishi M, Tanaka K, Morimoto H, Sakata K (2010). Calculation of the benchmark duration of shift work associated with the development of impaired glucose metabolism: a 14-year cohort study on 7104 male workers. Occup Environ Med.

[194] Lin YC, Hsiao TJ, Chen PC (2009). Shift work aggravates metabolic syndrome development among early-middle-aged males with elevated ALT. World J Gastroenterol.

[195] Pietroiusti A, Neri A, Somma G, Coppeta L, Iavicoli I, Bergamaschi A (2010). Incidence of metabolic syndrome among night-shift healthcare workers. Occup Environ Med.

[196] De BD, Van RM, Clays E, Kittel F, De BG, Braeckman L (2009). Rotating shift work and the metabolic syndrome: a prospective study. Int J Epidemiol.

[197] Eriksson AK, van den DM, Hilding A, Ostenson CG (2013). Work stress, sense of coherence, and risk of type 2 diabetes in a prospective study of middle-aged Swedish men and women. Diabetes Care.

[198] Pan A, Schernhammer ES, Sun Q, Hu FB (2011). Rotating night shift work and risk of type 2 diabetes: two prospective cohort studies in women. PLoS Med.

[199] Kroenke CH, Spiegelman D, Manson J, Schernhammer ES, Colditz GA, Kawachi I (2007). Work characteristics and incidence of type 2 diabetes in women. Am J Epidemiol.

[200] Suwazono Y, Sakata K, Okubo Y, Harada H, Oishi M, Kobayashi E (2006). Long-term longitudinal study on the relationship between alternating shift work and the onset of diabetes mellitus in male Japanese workers. J Occup Environ Med.

[201] Morikawa Y, Nakagawa H, Miura K, Soyama Y, Ishizaki M, Kido T (2005). Shift work and the risk of diabetes mellitus among Japanese male factory workers. Scand J Work Environ Health.

[202] Kennaway DJ, Varcoe TJ, Voultsios A, Boden MJ (2013). Global loss of bmal1 expression alters adipose tissue hormones, gene expression and glucose metabolism. PLoS One.

[203] Lee J, Moulik M, Fang Z, Saha P, Zou F, Xu Y (2013). Bmal1 and beta-cell clock are required for adaptation to circadian disruption, and their loss of function leads to oxidative stress-induced beta-cell failure in mice. Mol Cell Biol.

[204] Sadacca LA, Lamia KA, deLemos AS, Blum B, Weitz CJ (2011). An intrinsic circadian clock of the pancreas is required for normal insulin release and glucose homeostasis in mice. Diabetologia.

[205] Marcheva B, Ramsey KM, Buhr ED, Kobayashi Y, Su H, Ko CH (2010). Disruption of the clock components CLOCK and BMAL1 leads to hypoinsulinaemia and diabetes. Nature.

[206] Doi R, Oishi K, Ishida N (2010). CLOCK regulates circadian rhythms of hepatic glycogen synthesis through transcriptional activation of Gys2. J Biol Chem.

[207] Turek FW, Joshu C, Kohsaka A, Lin E, Ivanova G, McDearmon E (2005). Obesity and metabolic syndrome in circadian Clock mutant mice. Science.

[208] Rudic RD, McNamara P, Curtis AM, Boston RC, Panda S, Hogenesch JB (2004). BMAL1 and CLOCK, two essential components of the circadian clock, are involved in glucose homeostasis. PLoS Biol.

[209] Lamia KA, Storch KF, Weitz CJ (2008). Physiological significance of a peripheral tissue circadian clock. Proc Natl Acad Sci U S A.

[210] Sherman H, Genzer Y, Cohen R, Chapnik N, Madar Z, Froy O (2012). Timed high-fat diet resets circadian metabolism and prevents obesity. FASEB J.

[211] Leproult R, Holmback U, Van CE (2014). Circadian misalignment augments markers of insulin resistance and inflammation, independently of sleep loss. Diabetes.

[212] Lund J, Arendt J, Hampton SM, English J, Morgan LM (2001). Postprandial hormone and metabolic responses amongst shift workers in Antarctica. J Endocrinol.

[213] Esquirol Y, Bongard V, Ferrieres J, Verdier H, Perret B (2012). Shiftwork and higher pancreatic secretion: early detection of an intermediate state of insulin resistance?. Chronobiol Int.

[214] Gale JE, Cox HI, Qian J, Block GD, Colwell CS, Matveyenko AV (2011). Disruption of circadian rhythms accelerates development of diabetes through pancreatic beta-cell loss and dysfunction. J Biol Rhythms.

[215] Castanon-Cervantes O, Wu M, Ehlen JC, Paul K, Gamble KL, Johnson RL (2010). Dysregulation of inflammatory responses by chronic circadian disruption. J Immunol.

[216] Calle MC, Fernandez ML (2012). Inflammation and type 2 diabetes. Diabetes Metab.

[217] Young T, Skatrud J, Peppard PE (2004). Risk factors for obstructive sleep apnea in adults. JAMA.

[218] Jennum P, Riha RL (2009). Epidemiology of sleep apnoea/hypopnoea syndrome and sleep-disordered breathing. Eur Respir J.

[219] Punjabi NM (2008). The epidemiology of adult obstructive sleep apnea. Proc Am Thorac Soc.

[220] Baguet JP, Barone-Rochette G, Tamisier R, Levy P, Pepin JL (2012). Mechanisms of cardiac dysfunction in obstructive sleep apnea. Nat Rev Cardiol.

[221] Lopez-Jimenez F, Sert Kuniyoshi FH, Gami A, Somers VK (2008). Obstructive sleep apnea: implications for cardiac and vascular disease. Chest.

[222] Caples SM, Garcia-Touchard A, Somers VK (2007). Sleep-disordered breathing and cardiovascular risk. Sleep.

[223] Pamidi S, Aronsohn RS, Tasali E (2010). Obstructive sleep apnea: role in the risk and severity of diabetes. Best Pract Res Clin Endocrinol Metab.

[224] Punjabi NM (2009). Do sleep disorders and associated treatments impact glucose metabolism?. Drugs.

[225] Levy P, Bonsignore MR, Eckel J (2009). Sleep, sleep-disordered breathing and metabolic consequences. Eur Respir J.

[226] Jun J, Polotsky VY (2009). Metabolic consequences of sleep-disordered breathing. ILAR J.

[227] Seicean S, Kirchner HL, Gottlieb DJ, Punjabi NM, Resnick H, Sanders M (2008). Sleep-disordered breathing and impaired glucose metabolism in normal-weight and overweight/obese individuals: the Sleep Heart Health Study. Diabetes Care.

[228] Punjabi NM, Shahar E, Redline S, Gottlieb DJ, Givelber R, Resnick HE (2004). Sleep-disordered breathing, glucose intolerance, and insulin resistance: the Sleep Heart Health Study. Am J Epidemiol.

[229] McArdle N, Hillman D, Beilin L, Watts G (2007). Metabolic risk factors for vascular disease in obstructive sleep apnea: a matched controlled study. Am J Respir Crit Care Med.

[230] Ip MS, Lam B, Ng MM, Lam WK, Tsang KW, Lam KS (2002). Obstructive sleep apnea is independently associated with insulin resistance. Am J Respir Crit Care Med.

[231] Pamidi S, Tasali E (2012). Obstructive sleep apnea and type 2 diabetes: is there a link?. Front Neurol.

[232] Borel AL, Monneret D, Tamisier R, Baguet JP, Faure P, Levy P (2013). The severity of nocturnal hypoxia but not abdominal adiposity is associated with insulin resistance in non-obese men with sleep apnea. PLoS One.

[233] Lindberg E, Theorell-Haglow J, Svensson M, Gislason T, Berne C, Janson C (2012). Sleep apnea and glucose metabolism: a long-term follow-up in a community-based sample. Chest.

[234] Botros N, Concato J, Mohsenin V, Selim B, Doctor K, Yaggi HK (2009). Obstructive sleep apnea as a risk factor for type 2 diabetes. Am J Med.

[235] Marshall NS, Wong KK, Phillips CL, Liu PY, Knuiman MW, Grunstein RR (2009). Is sleep apnea an independent risk factor for prevalent and incident diabetes in the Busselton Health Study?. J Clin Sleep Med.

[236] Reichmuth KJ, Austin D, Skatrud JB, Young T (2005). Association of sleep apnea and type II diabetes: a population-based study. Am J Respir Crit Care Med.

[237] Wang X, Bi Y, Zhang Q, Pan F (2013). Obstructive sleep apnoea and the risk of type 2 diabetes: a meta-analysis of prospective cohort studies. Respirology.

[238] Kribbs NB, Pack AI, Kline LR, Smith PL, Schwartz AR, Schubert NM (1993). Objective measurement of patterns of nasal CPAP use by patients with obstructive sleep apnea. Am Rev Respir Dis.

[239] Grimaldi D, Beccuti G, Touma C, Van CE, Mokhlesi B (2014). Association of obstructive sleep apnea in rapid eye movement sleep with reduced glycemic control in type 2 diabetes: therapeutic implications. Diabetes Care.

[240] Babu AR, Herdegen J, Fogelfeld L, Shott S, Mazzone T (2005). Type 2 diabetes, glycemic control, and continuous positive airway pressure in obstructive sleep apnea. Arch Intern Med.

[241] Sookoian S, Pirola CJ (2013). Obstructive sleep apnea is associated with fatty liver and abnormal liver enzymes: a meta-analysis. Obes Surg.

[242] Polotsky VY, Patil SP, Savransky V, Laffan A, Fonti S, Frame LA (2009). Obstructive sleep apnea, insulin resistance, and steatohepatitis in severe obesity. Am J Respir Crit Care Med.

[243] Mishra P, Nugent C, Afendy A, Bai C, Bhatia P, Afendy M (2008). Apnoeic-hypopnoeic episodes during obstructive sleep apnoea are associated with histological nonalcoholic steatohepatitis. Liver Int.

[244] ron-Wisnewsky J, Minville C, Tordjman J, Levy P, Bouillot JL, Basdevant A (2012). Chronic intermittent hypoxia is a major trigger for non-alcoholic fatty liver disease in morbid obese. J Hepatol.

[245] Tatsumi K, Saibara T (2005). Effects of obstructive sleep apnea syndrome on hepatic steatosis and nonalcoholic steatohepatitis. Hepatol Res.

[246] Minville C, Hilleret MN, Tamisier R, ron-Wisnewsky J, Clement K, Trocme C (2014). Nonalcoholic fatty liver disease, nocturnal hypoxia, and endothelial function in patients with sleep apnea. Chest.

[247] Sundaram SS, Sokol RJ, Capocelli KE, Pan Z, Sullivan JS, Robbins K (2014). Obstructive sleep apnea and hypoxemia are associated with advanced liver histology in pediatric nonalcoholic fatty liver disease. J Pediatr.

[248] Polotsky VY, Li J, Punjabi NM, Rubin AE, Smith PL, Schwartz AR (2003). Intermittent hypoxia increases insulin resistance in genetically obese mice. J Physiol.

[249] Drager LF, Yao Q, Hernandez KL, Shin MK, Bevans-Fonti S, Gay J (2013). Chronic intermittent hypoxia induces atherosclerosis via activation of adipose angiopoietin-like 4. Am J Respir Crit Care Med.

[250] Polak J, Shimoda LA, Drager LF, Undem C, McHugh H, Polotsky VY (2013). Intermittent hypoxia impairs glucose homeostasis in C57BL6/J mice: partial improvement with cessation of the exposure. Sleep.

[251] Drager LF, Li J, Reinke C, Bevans-Fonti S, Jun JC, Polotsky VY (2011). Intermittent hypoxia exacerbates metabolic effects of diet-induced obesity. Obesity (Silver Spring).

[252] Iiyori N, Alonso LC, Li J, Sanders MH, Garcia-Ocana A, O'Doherty RM (2007). Intermittent hypoxia causes insulin resistance in lean mice independent of autonomic activity. Am J Respir Crit Care Med.

[253] Chen L, Cao ZL, Han F, Gao ZC, He QY (2010). Chronic intermittent hypoxia from pedo-stage decreases glucose transporter 4 expression in adipose tissue and causes insulin resistance. Chin Med J (Engl).

[254] Fenik VB, Singletary T, Branconi JL, Davies RO, Kubin L (2012). Glucoregulatory consequences and cardiorespiratory parameters in rats exposed to chronic-intermittent hypoxia: effects of the duration of exposure and losartan. Front Neurol.

[255] Louis M, Punjabi NM (2009). Effects of acute intermittent hypoxia on glucose metabolism in awake healthy volunteers. J Appl Physiol.

[256] Rosa DP, Martinez D, Picada JN, Semedo JG, Marroni NP (2011). Hepatic oxidative stress in an animal model of sleep apnoea: effects of different duration of exposure. Comp Hepatol.

[257] Savransky V, Nanayakkara A, Vivero A, Li J, Bevans S, Smith PL (2007). Chronic intermittent hypoxia predisposes to liver injury. Hepatology.

[258] Savransky V, Bevans S, Nanayakkara A, Li J, Smith PL, Torbenson MS (2007). Chronic intermittent hypoxia causes hepatitis in a mouse model of diet-induced fatty liver. Am J Physiol Gastrointest Liver Physiol.

[259] Chen XY, Zeng YM, Zhang YX, Wang WY, Wu RH (2013). Effect of chronic intermittent hypoxia on theophylline metabolism in mouse liver. Chin Med J (Engl).

[260] Li J, Thorne LN, Punjabi NM, Sun CK, Schwartz AR, Smith PL (2005). Intermittent hypoxia induces hyperlipidemia in lean mice. Circ Res.

[261] Savransky V, Nanayakkara A, Li J, Bevans S, Smith PL, Rodriguez A (2007). Chronic intermittent hypoxia induces atherosclerosis. Am J Respir Crit Care Med.

[262] Li J, Bosch-Marce M, Nanayakkara A, Savransky V, Fried SK, Semenza GL (2006). Altered metabolic responses to intermittent hypoxia in mice with partial deficiency of hypoxia-inducible factor-1alpha. Physiol Genomics.

[263] da Rosa DP, Forgiarini LF, Baronio D, Feijo CA, Martinez D, Marroni NP (2012). Simulating sleep apnea by exposure to intermittent hypoxia induces inflammation in the lung and liver. Mediators Inflamm.

[264] Jun J, Savransky V, Nanayakkara A, Bevans S, Li J, Smith PL (2008). Intermittent hypoxia has organ-specific effects on oxidative stress. Am J Physiol Regul Integr Comp Physiol.

[265] Tanne F, Gagnadoux F, Chazouilleres O, Fleury B, Wendum D, Lasnier E (2005). Chronic liver injury during obstructive sleep apnea. Hepatology.

[266] Pastoris O, Gorini A, Vercesi L, Taglietti M, Dossena M (1985). Modification of the skeletal muscle energy metabolism induced by intermittent normobaric hypoxia and treatment with biological pyrimidines. Farmaco Sci.

[267] Carreras A, Kayali F, Zhang J, Hirotsu C, Wang Y, Gozal D (2012). Metabolic effects of intermittent hypoxia in mice: steady versus high-frequency applied hypoxia daily during the rest period. Am J Physiol Regul Integr Comp Physiol.

[268] Pallayova M, Lazurova I, Donic V (2011). Hypoxic damage to pancreatic beta cells–the hidden link between sleep apnea and diabetes. Med Hypotheses.

[269] Pallayova M, Steele KE, Magnuson TH, Schweitzer MA, Hill NR, Bevans-Fonti S (2010). Sleep apnea predicts distinct alterations in glucose homeostasis and biomarkers in obese adults with normal and impaired glucose metabolism. Cardiovasc Diabetol.

[270] Yokoe T, Alonso LC, Romano LC, Rosa TC, O'Doherty RM, Garcia-Ocana A (2008). Intermittent hypoxia reverses the diurnal glucose rhythm and causes pancreatic beta-cell replication in mice. J Physiol.

[271] Xu J, Long YS, Gozal D, Epstein PN (2009). Beta-cell death and proliferation after intermittent hypoxia: role of oxidative stress. Free Radic Biol Med.

[272] Wang N, Khan SA, Prabhakar NR, Nanduri J (2013). Impairment of pancreatic beta-cell function by chronic intermittent hypoxia. Exp Physiol.

[273] Ota H, Tamaki S, Itaya-Hironaka A, Yamauchi A, Sakuramoto-Tsuchida S, Morioka T (2012). Attenuation of glucose-induced insulin secretion by intermittent hypoxia via down-regulation of CD38. Life Sci.

[274] Delarue J, Magnan C (2007). Free fatty acids and insulin resistance. Curr Opin Clin Nutr Metab Care.

[275] Jun J, Reinke C, Bedja D, Berkowitz D, Bevans-Fonti S, Li J (2010). Effect of intermittent hypoxia on atherosclerosis in apolipoprotein E-deficient mice. Atherosclerosis.

[276] Jun JC, Drager LF, Najjar SS, Gottlieb SS, Brown CD, Smith PL (2011). Effects of sleep apnea on nocturnal free fatty acids in subjects with heart failure. Sleep.

[277] Poulain L, Thomas A, Rieusset J, Casteilla L, Levy P, Arnaud C (2014). Visceral white fat remodelling contributes to intermittent hypoxia-induced atherogenesis. Eur Respir J.

[278] Yao Q, Shin MK, Jun JC, Hernandez KL, Aggarwal NR, Mock JR (2013). Effect of chronic intermittent hypoxia on triglyceride uptake in different tissues. J Lipid Res.

[279] Magalang UJ, Cruff JP, Rajappan R, Hunter MG, Patel T, Marsh CB (2009). Intermittent hypoxia suppresses adiponectin secretion by adipocytes. Exp Clin Endocrinol Diabetes.

[280] Borst SE, Conover CF, Bagby GJ (2005). Association of resistin with visceral fat and muscle insulin resistance. Cytokine.

[281] Carlson JT, Hedner J, Elam M, Ejnell H, Sellgren J, Wallin BG (1993). Augmented resting sympathetic activity in awake patients with obstructive sleep apnea. Chest.

[282] Somers VK, Dyken ME, Mark AL, Abboud FM (1993). Sympathetic-nerve activity during sleep in normal subjects. N Engl J Med.

[283] Tamisier R, Pepin JL, Remy J, Baguet JP, Taylor JA, Weiss JW (2011). 14 nights of intermittent hypoxia elevate daytime blood pressure and sympathetic activity in healthy humans. Eur Respir J.

[284] Prabhakar NR, Kumar GK (2010). Mechanisms of sympathetic activation and blood pressure elevation by intermittent hypoxia. Respir Physiol Neurobiol.

[285] Xing T, Pilowsky PM, Fong AY (2014). Mechanism of sympathetic activation and blood pressure elevation in humans and animals following acute intermittent hypoxia. Prog Brain Res.

[286] Prabhakar NR, Kumar GK, Peng YJ (2012). Sympatho-adrenal activation by chronic intermittent hypoxia. J Appl Physiol (1985).

[287] Ahren B (2000). Autonomic regulation of islet hormone secretion–implications for health and disease. Diabetologia.

[288] Shimazu T (1996). Innervation of the liver and glucoregulation: roles of the hypothalamus and autonomic nerves. Nutrition.

[289] Youngstrom TG, Bartness TJ (1995). Catecholaminergic innervation of white adipose tissue in Siberian hamsters. Am J Physiol.

[290] Deibert DC, DeFronzo RA (1980). Epinephrine-induced insulin resistance in man. J Clin Invest.

[291] Ribeiro MJ, Sacramento JF, Gonzalez C, Guarino MP, Monteiro EC, Conde SV (2013). Carotid body denervation prevents the development of insulin resistance and hypertension induced by hypercaloric diets. Diabetes.

[292] Shin MK, Yao Q, Jun JC, Bevans-Fonti S, Yoo DY, Han W (2014). Carotid body denervation prevents fasting hyperglycemia during chronic intermittent hypoxia. J Appl Physiol (1985).

[293] Prigge WF, Grande F (1971). Effects of glucagon, epinephrine and insulin on in vitro lipolysis of adipose tissue from mammals and birds. Comp Biochem Physiol B.

[294] Goodridge AG, Ball EG (1965). Studies on the metabolism of adipose tissue. 18. In vitro effects of insulin, epinephrine and glucagon on lipolysis and glycolysis in pigeon adipose tissue. Comp Biochem Physiol.

[295] Bartness TJ, Shrestha YB, Vaughan CH, Schwartz GJ, Song CK (2010). Sensory and sympathetic nervous system control of white adipose tissue lipolysis. Mol Cell Endocrinol.

[296] Lafontan M, Berlan M (1995). Fat cell alpha 2-adrenoceptors: the regulation of fat cell function and lipolysis. Endocr Rev.

[297] Yi CX, La Fleur SE, Fliers E, Kalsbeek A (1802). The role of the autonomic nervous liver innervation in the control of energy metabolism. Biochim Biophys Acta.

[298] Lambert GW, Straznicky NE, Lambert EA, Dixon JB, Schlaich MP (2010). Sympathetic nervous activation in obesity and the metabolic syndrome–causes, consequences and therapeutic implications. Pharmacol Ther.

[299] Jun JC, Shin MK, Devera R, Yao Q, Mesarwi O, Bevans-Fonti S (2014). Intermittent hypoxia-induced glucose intolerance is abolished by α-adrenergic blockade or adrenal medullectomy. Am J Physiol Endocrinol Metab.

[300] Shin MK, Han W, Bevans-Fonti S, Jun JC, Punjabi NM, Polotsky VY (2014). The effect of adrenal medullectomy on metabolic responses to chronic intermittent hypoxia. Respir Physiol Neurobiol.

[301] Lesser DJ, Bhatia R, Tran WH, Oliveira F, Ortega R, Keens TG (2012). Sleep fragmentation and intermittent hypoxemia are associated with decreased insulin sensitivity in obese adolescent Latino males. Pediatr Res.

[302] Stamatakis KA, Punjabi NM (2010). Effects of sleep fragmentation on glucose metabolism in normal subjects. Chest.

[303] Tasali E, Leproult R, Ehrmann DA, Van CE (2008). Slow-wave sleep and the risk of type 2 diabetes in humans. Proc Natl Acad Sci U S A.

[304] Gonnissen HK, Hursel R, Rutters F, Martens EA, Westerterp-Plantenga MS (2013). Effects of sleep fragmentation on appetite and related hormone concentrations over 24 h in healthy men. Br J Nutr.

[305] Pogach MS, Punjabi NM, Thomas N, Thomas RJ (2012). Electrocardiogram-based sleep spectrogram measures of sleep stability and glucose disposal in sleep disordered breathing. Sleep.

[306] Trento M, Broglio F, Riganti F, Basile M, Borgo E, Kucich C (2008). Sleep abnormalities in type 2 diabetes may be associated with glycemic control. Acta Diabetol.

[307] Ekstedt M, Akerstedt T, Soderstrom M (2004). Microarousals during sleep are associated with increased levels of lipids, cortisol, and blood pressure. Psychosom Med.

[308] van den Berg JF, Knvistingh NA, Tulen JH, Hofman A, Witteman JC, Miedema HM (2008). Actigraphic sleep duration and fragmentation are related to obesity in the elderly: the Rotterdam Study. Int J Obes (Lond).

[309] Sawamoto R, Nozaki T, Furukawa T, Tanahashi T, Morita C, Hata T (2014). Higher sleep fragmentation predicts a lower magnitude of weight loss in overweight and obese women participating in a weight-loss intervention. Nutr Diabetes.

[310] Baud MO, Magistretti PJ, Petit JM (2013). Sustained sleep fragmentation affects brain temperature, food intake and glucose tolerance in mice. J Sleep Res.

[311] Barf RP, Meerlo P, Scheurink AJ (2010). Chronic sleep disturbance impairs glucose homeostasis in rats. Int J Endocrinol.

[312] Wang Y, Carreras A, Lee S, Hakim F, Zhang SX, Nair D (2014). Chronic sleep fragmentation promotes obesity in young adult mice. Obesity (Silver Spring).

[313] Zhang SX, Khalyfa A, Wang Y, Carreras A, Hakim F, Neel BA (2014). Sleep fragmentation promotes NADPH oxidase 2-mediated adipose tissue inflammation leading to insulin resistance in mice. Int J Obes (Lond).

[314] Gharib SA, Khalyfa A, Abdelkarim A, Bhushan B, Gozal D (2012). Integrative miRNA-mRNA profiling of adipose tissue unravels transcriptional circuits induced by sleep fragmentation. PLoS One.

[315] Khalyfa A, Wang Y, Zhang SX, Qiao Z, Abdelkarim A, Gozal D (2014). Sleep fragmentation in mice induces nicotinamide adenine dinucleotide phosphate oxidase 2-dependent mobilization, proliferation, and differentiation of adipocyte progenitors in visceral white adipose tissue. Sleep.

[316] Khalyfa A, Mutskov V, Carreras A, Khalyfa AA, Hakim F, Gozal D (2014). Sleep fragmentation during late gestation induces metabolic perturbations and epigenetic changes in adiponectin gene expression in male adult offspring mice. Diabetes.

[317] Herzog N, Jauch-Chara K, Hyzy F, Richter A, Friedrich A, Benedict C (2013). Selective slow wave sleep but not rapid eye movement sleep suppression impairs morning glucose tolerance in healthy men. Psychoneuroendocrinology.

[318] Hursel R, Rutters F, Gonnissen HK, Martens EA, Westerterp-Plantenga MS (2011). Effects of sleep fragmentation in healthy men on energy expenditure, substrate oxidation, physical activity, and exhaustion measured over 48 h in a respiratory chamber. Am J Clin Nutr.

[319] Feupe SF, Frias PF, Mednick SC, McDevitt EA, Heintzman ND (2013). Nocturnal continuous glucose and sleep stage data in adults with type 1 diabetes in real-world conditions. J Diabetes Sci Technol.

[320] Theorell-Haglow J, Berne C, Janson C, Sahlin C, Lindberg E (2010). Associations between short sleep duration and central obesity in women. Sleep.

[321] Liu X, Forbes EE, Ryan ND, Rofey D, Hannon TS, Dahl RE (2008). Rapid eye movement sleep in relation to overweight in children and adolescents. Arch Gen Psychiatry.

[322] Horne J (2009). REM sleep, energy balance and 'optimal foraging'. Neurosci Biobehav Rev.

[323] Fontvieille AM, Rising R, Spraul M, Larson DE, Ravussin E (1994). Relationship between sleep stages and metabolic rate in humans. Am J Physiol.

